# History biases reveal novel dissociations between perceptual and metacognitive decision-making

**DOI:** 10.1167/jov.23.5.14

**Published:** 2023-05-18

**Authors:** Christopher S. Y. Benwell, Rachael Beyer, Francis Wallington, Robin A. A. Ince

**Affiliations:** 1 Division of Psychology, School of Humanities, Social Sciences and Law, University of Dundee, Dundee, UK; 2 School of Psychology and Neuroscience, University of Glasgow, Glasgow, UK; 3 School of Psychology and Neuroscience, University of Glasgow, Glasgow, UK; 4 School of Psychology and Neuroscience, University of Glasgow, Glasgow, UK; 5 School of Psychology and Neuroscience, University of Glasgow, Glasgow, UK

**Keywords:** metacognition, history bias, perception, serial dependence, computational modeling

## Abstract

Human decision-making and self-reflection often depend on context and internal biases. For instance, decisions are often influenced by preceding choices, regardless of their relevance. It remains unclear how choice history influences different levels of the decision-making hierarchy. We used analyses grounded in information and detection theories to estimate the relative strength of perceptual and metacognitive history biases and to investigate whether they emerge from common/unique mechanisms. Although both perception and metacognition tended to be biased toward previous responses, we observed novel dissociations that challenge normative theories of confidence. Different evidence levels often informed perceptual and metacognitive decisions within observers, and response history distinctly influenced first- (perceptual) and second- (metacognitive) order decision-parameters, with the metacognitive bias likely to be strongest and most prevalent in the general population. We propose that recent choices and subjective confidence represent heuristics, which inform first- and second-order decisions in the absence of more relevant evidence.

## Introduction

Human knowledge of the external world and of internal cognitive processes is often biased and incomplete (Wilson & Dunn, 2004; Johansson, Hall, Sikstrom, & Olsson, 2005; Johnson & Fowler, 2011). When decisions are made about sensory input (i.e., Is a target present?), we can distinguish between objective accuracy (perceptual sensitivity) and how accurate one is in judging their own performance (metacognitive sensitivity) (Galvin, Podd, Drga, & Whitmore, 2003; Maniscalco & Lau, 2012). Metacognitive sensitivity can be quantified by comparing subjective confidence to objective accuracy (Fleming & Lau, 2014). Although accuracy and confidence usually correlate, metacognitive performance differs widely across individuals (Johnson & Fowler, 2011; Fleming, Thomas, & Dolan, 2010; Peters et al., 2017; Shekhar & Rahnev, 2021) with important consequences in everyday life. For instance, insight modulates learning, adaptive decision-making, error monitoring, and exploration (van den Berg, Zylberberg, Kiani, Shadlen, & Wolpert, 2016; Desender, Boldt, & Yeung, 2018; Yeung & Summerfield, 2012; Bahrami et al., 2012; Folke, Jacobsen, Fleming, & De Martino, 2016). In fact, impaired metacognition is associated with many neuropsychiatric disorders (David, Bedford, Wiffen, & Gilleen, 2012) and sub-clinical symptom dimensions (Rouault, Seow, Gillan, & Fleming, 2018; Benwell, Mohr, Wallberg, Kouadio, & Ince, 2022).

Even in healthy individuals, perceptual and metacognitive decisions not only depend on the immediately available evidence but also on recent experiences and choices. For instance, when similar stimuli are serially presented, perceptual decisions are often biased toward responses or stimuli on preceding trials, a phenomenon known as choice history bias (Urai, Braun, & Donner, 2017; Braun, Urai, & Donner, 2018; Bonaiuto, Berker, & Bestmann, 2016; Abrahamyan, Silva, Dakin, Carandini, & Gardner, 2016; Urai, De Gee, Tsetsos, & Donner, 2019; Fernberger, 1920) or serial dependence (Fritsche, Mostert, & de Lange, 2017; Fischer & Whitney, 2014; Bliss, Sun, & D'Esposito, 2017; Liberman, Fischer, & Whitney, 2014; John-Saaltink, Kok, Lau, & De Lange, 2016; Pascucci et al., 2019; Pascucci et al., 2023). Although this mechanism may generally be adaptive (because recent experience usually predicts upcoming input), it can also lead to non-veridical decisions (Fischer & Whitney, 2014; Kiyonaga, Scimeca, Bliss, & Whitney, 2017; Cicchini, Mikellidou, & Burr, 2018; Manassi, Liberman, Chaney, & Whitney, 2017). Interestingly, serial dependence has also been reported for subjective confidence reports (Rahnev, Koizumi, McCurdy, D'Esposito, & Lau, 2015; Mei, Rahnev, & Soto, 2023), and the level of confidence on the preceding trial has been suggested to modulate perceptual history bias, with repetition more likely when preceding confidence was high (Urai et al., 2017; Braun et al., 2018; Samaha, Switzky, & Postle, 2019; Bosch, Fritsche, Ehinger, & de Lange, 2020). These reports suggest the existence of an intimate link between perception and metacognition in the formation of history biases. However, the exact nature of this relationship, and the relative strength and source of each bias, remain unclear.

Using both model-based and nonparametric analyses, we observed history biases in both perceptual responses and ratings of confidence, but we show that the metacognitive history bias is stronger and likely to be most prevalent in the general population. Computational modeling revealed intriguing dissociations between perceptual and metacognitive decision-making parameters. For instance, perceptual choice alternation (disengagement from hysteresis) was associated with increased perceptual sensitivity but reduced metacognitive insight. Overall performance closely matched predictions from recently proposed computational models of decision-making and confidence (Kepecs, Uchida, Zariwala, & Mainen, 2008; Sanders, Hangya, & Kepecs, 2016; Hebart, Schriever, Donner, & Haynes, 2016; Masset, Ott, Lak, Hirokawa, J., Kepecs, 2020). However, we crucially demonstrate that both perceptual and metacognitive decision criteria are not fixed; they fluctuate from moment to moment and are biased by recent choices. Accurate models of subjective confidence must go beyond a normative account to capture suboptimal metacognitive performance driven by irrelevant factors such as preceding confidence reports.

## Materials and methods

### Participants

Forty-three healthy human observers participated in the study. All reported normal or corrected-to-normal vision. The sample size was chosen to ensure statistical power equal to or higher than previous studies that detected choice history bias in both perceptual decisions (Urai et al., 2017; Braun et al., 2018; Bonaiuto et al., 2016; Abrahamyan et al., 2016) and confidence ratings (Rahnev et al., 2015). Because of poor psychophysical performance (explained in the *Data Exclusion* section), six participants were excluded from the analysis, leaving a total number of 37 participants (26 female/11 male aged from 18 to 38 years [*M* = 25.23, *SD* = 3.95]). The study adhered to the Declaration of Helsinki and was approved by the Ethics Committee of the College of Science and Engineering at the University of Glasgow, and all participants gave their informed consent. No monetary reward was given to participants for taking part, although undergraduate students could receive course credits for their participation.

### Stimuli and task

**Figure 1.**
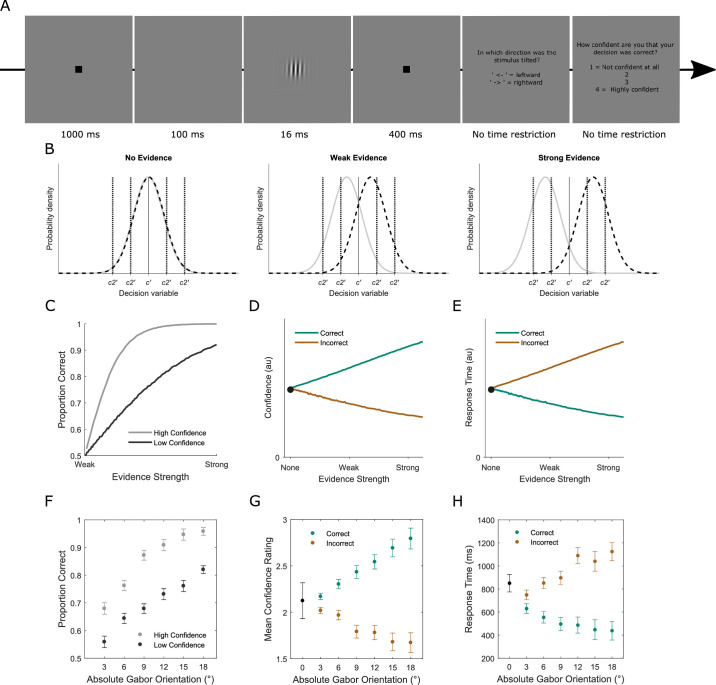
**(A)** Behavioral task. On each trial, a Gabor orientation discrimination judgement was made followed by a confidence report (scale of 1 to 4, where 1 represented “not confident at all” and 4 represented “highly confident”). **(B)** Computational model of decision making and confidence in a 2-AFC task. The probability density functions represent distributions of internal responses (decision variables (DV)) across repeated presentations of the generative stimulus. On each trial, the DV is drawn from one of these distributions and compared with a decision criterion (*c’*: solid black vertical line) to reach a binary choice. The level of confidence in the choice is then reflected in the absolute distance of the DV from *c’*. When a discrete confidence rating scale is employed, the level of reported confidence is defined by where the DV falls with respect to the type-2 criteria (*c2ʹ_1_, c2ʹ_2_*, … *c2ʹ_(N−1)_*: dashed vertical black lines), where *N* indexes the number of possible ratings. The type-2 (or confidence) criteria (*c2ʹ*) govern how far the DV must be from *cʹ* before an individual is willing to report a given level of confidence. A confidence rating of k will be given if the DV falls in the interval (*c2ʹ_k−1_, c2ʹ_k_*). The relative separation on the x-axis of the two distributions indexes the level of evidence available for the decision. The model is plotted for three levels of overall decision evidence: none (left panel), weak (center panel) and strong (right panel). **(C)** Model-based prediction of the relationship between decision accuracy and evidence strength as a function of confidence level. **(D)** Predicted relationship between decision confidence and evidence strength as a function of accuracy. **(E)** Predicted relationship between response time and evidence strength as a function of accuracy. These model-based predictions were all confirmed in the data. **(F)** Relationship between decision accuracy and absolute Gabor orientation as a function of confidence level. Note that data are not presented for the 0° orientation because there was no correct response here. **(G)** Relationship between decision confidence and absolute Gabor orientation as a function of accuracy. **(H)** Relationship between response time and evidence strength as a function of accuracy.

The stimuli were Gabor patches (windowed sine wave gratings: 96 × 96 pixels [2.54 × 2.45 cm]) presented at the center of the screen. The Gabor patches had a peak contrast of 100% Michelson, a spatial frequency of 3.7 cycles per degree and a 0.3° standard deviation Gaussian contrast envelope. At a viewing distance of 57 cm (fixed using a chinrest), Gabor patches subtended 2.55° of visual angle. On each trial, the stimulus would appear at a random angle that ranged from −18° to 18° relative to vertical at intervals of 3° (including 0°). The monitor used to present the stimuli had a display refresh rate of 60 Hz and screen resolution of 1920 × 1080 pixels. The software used to implement the task was E-prime 2.0 and participants made responses using a QWERTY keyboard. Each trial began with a fixation point displayed at the center of the screen for 1000 ms (see Figure 1A). Following this, a Gabor patch appeared at a random orientation in the center of the screen for a duration of 16 ms. After the stimulus disappeared, the participant viewed the fixation point for 400 ms, before being instructed to indicate whether they perceived the top of the Gabor patch to be tilted in a “leftward” or “rightward” direction relative to vertical (two-alternative forced choice), by responding with the left and right arrow keys, respectively. Participants were not informed as to the accuracy of their choice, and no time limit was enforced. Immediately after responding, participants were presented with a second decision regarding their confidence about the perceptual choice they had just made. Participants were asked to rate their confidence on a scale of 1 to 4, where 1 represented “not confident at all” and 4 represented “highly confident,” using the corresponding digit keys on the keyboard. Immediately after making this response the central fixation point reappeared indicating the beginning of the next trial. A short practice block (12 trials), including only the most extreme angles (−18°, 18°) and with accuracy feedback on each trial, was performed to familiarize participants with the task. In the full experiment, each of the 13 orientations was presented 32 times in a randomized order, amounting to 416 trials in total. The experimental session lasted approximately 30 minutes.

### Quantifying the psychometric function

To model Gabor orientation discrimination performance, cumulative logistic PFs were fit to the data using a Maximum Likelihood criterion (Prins & Kingdom, 2018). The dependent measure was the proportion of trials on which the participant indicated that the Gabor appeared to be oriented “rightward”, and the independent measure was the true orientation of the Gabor. The logistic function is described by the following:

$$\begin{array}{c} f\left(x;\delta ,\alpha \right)=\;\gamma +\left(1-\gamma -\lambda \right)\times \left(\frac{1}{1+e^{\left(-1\delta \left(x-\alpha \right)\right)}}\right) \end{array}$$

where *x* is the tested Gabor orientation, *δ* is the subjective threshold (location on the x-axis corresponding to 50% “left”/50% “right” responses), and *α* is the slope of the rising curve (indexing visual sensitivity). Both *λ* and *γ* represent the probability of stimulus independent lapses and were fixed at 0.02.

### Data exclusion

The PF threshold and slope parameters were used to formally detect outliers in the dataset. Any participant who met any one of the following two criteria for the overall PF fit to their entire dataset was excluded from further analysis: (1) a threshold value over 3 median absolute deviations from the overall group median or (2) a slope value over 3 median absolute deviations from the overall group median. This led to a total of six participants being excluded, and, hence, 37 participants were entered into the final inferential analyses.

### Quantifying perceptual choice history bias

**Figure 2.**
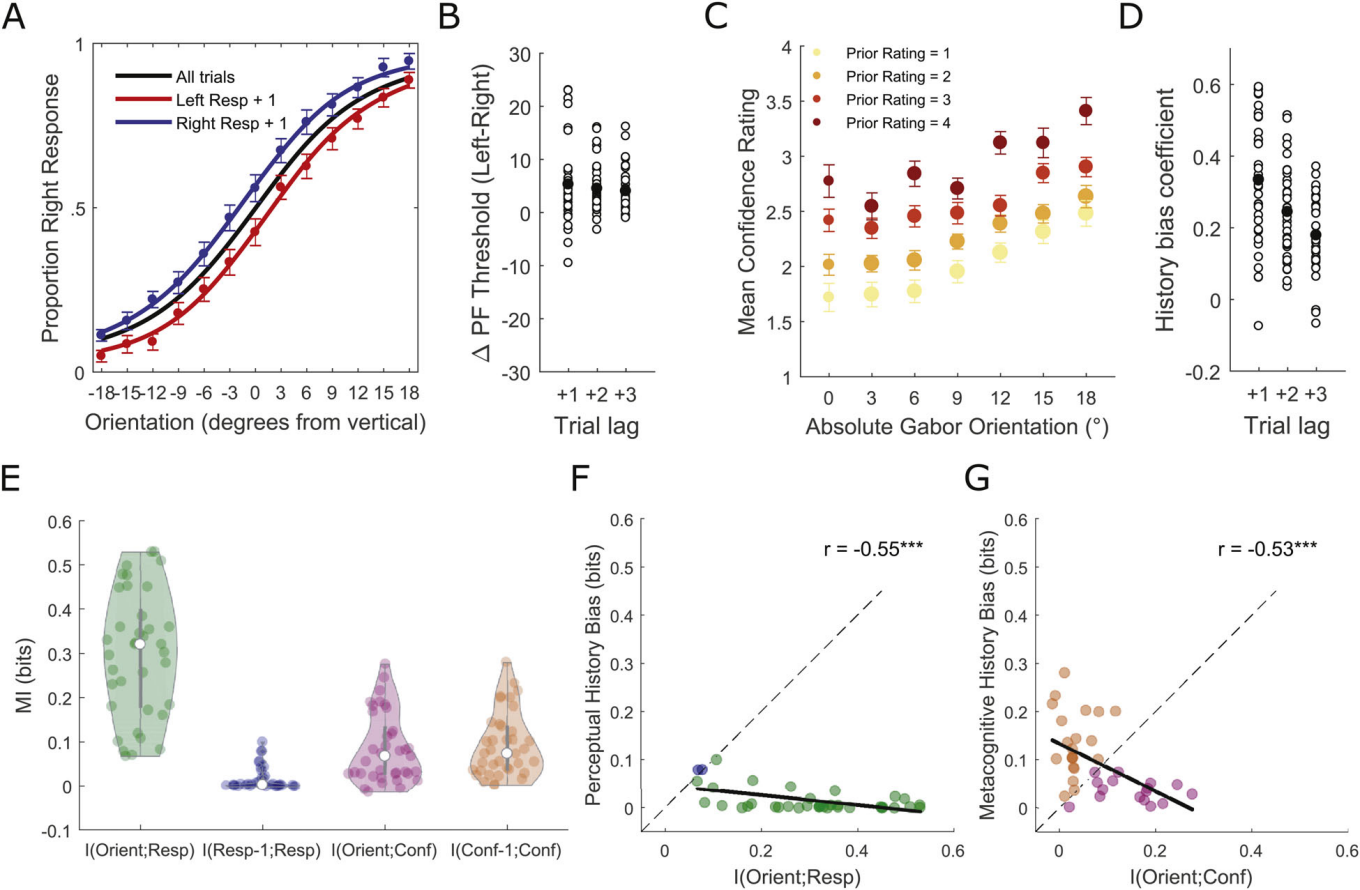
**(A)** Choice history biases perceptual decisions. Group-averaged PFs across all trials and conditioned on the previous perceptual choice. **(B)** Scatterplot of single-participant differences in PF threshold between “post left choice” and “post right choice” trials at trial lags of 1, 2 and 3 (black filled dots represent the group means). Positive values index a bias in favor of repeating the previous choice and negative values index a bias in favor of alternating the previous choice. Note that the perceptual bias was no longer statistically significant at trial lag +4. **(C)** Choice history biases confidence ratings. Group-averaged confidence ratings as a function of absolute Gabor orientation on the current trial and rating on the previous trial. The size of the dots indexes the relative number of trials contributing to the group average as this was not uniform across orientations and previous ratings. **(D)** Scatterplot of single-participant regression coefficients for the linear relationship between confidence on the previous and current trials at lags of 1, 2 and 3. Positive values index that “high”/“low” confidence ratings were more likely following “high”/“low” ratings respectively. Note that the confidence bias remained statistically significant up to trial lag 25. **(E)** Non-parametric within-participant MI analysis quantified the dependence between evidence presented on each trial (i.e., the orientation of the Gabor) and the perceptual responses/confidence ratings and, on the same effect size scale, the choice history biases in both perceptual responses and confidence ratings. **(F)** The relationship between perceptual choice history bias and the trial-by-trial influence of evidence on the perceptual decision. The influence of evidence was stronger in most participants (green dots) than the influence of choice history (blue dots). **(G)** The relationship between metacognitive choice history bias and the trial-by-trial influence of evidence on confidence ratings. There were relatively even sub-groups of participants for whom current evidence dominated confidence judgements (pink dots) vs those for whom choice history dominated confidence judgements (orange dots). Solid black lines represent least-squares regression slopes. All error bars represent within-subject ± standard error (SEM). ***p < 0.001.

To measure perceptual choice history bias, the data within each participant were split into two bins: one containing all trials that followed a leftward orientation response on the previous trial (“post left response”) and the other containing trials that followed a rightward orientation response on the previous trial (“post right response”). The PF was fit separately to data from these subsets of trials (Figure 2A). From the resulting fits, the threshold and slope were retrieved. This was done separately for trial lags of 1, 2, and 3. The difference in PF threshold between “post left” and “post right” responses indexed the strength and direction of perceptual choice history bias (Figure 2B). If positive choice history bias (i.e., tendency to repeat previous choices) heavily influences the orientation judgements, then the group-averaged psychometric curves conditioned separately on “post left response” and “post right response” trials will be shifted horizontally on the *x*-axis in relation to one another. To formally test this, a repeated-measures *t* test was performed to compare the PF thresholds between “post left response” trials and “post right response” trials. This analysis was also performed separately for “post high confidence” and “post low confidence” trials, respectively (Supplementary Figure S1: see *SDT parameter analyses* section below for division of confidence bins).

### Quantifying metacognitive choice history bias

Measuring history bias of metacognitive decisions required a different analytical approach. If positive metacognitive choice history bias occurs (Rahnev et al., 2015), then confidence ratings will be more likely to be high after a high confidence rating and low after a low confidence rating, regardless of the level of external evidence (i.e., absolute Gabor orientation) (see Figure 2C). To statistically test this, linear regression was performed between absolute Gabor orientation and mean confidence ratings separately for post 1, 2, 3, and 4 rating trials in each participant. Subsequently, linear regression was then performed between the previous confidence rating (1, 2, 3, 4) and the intercepts of the orientation-confidence regressions, and the resulting within-participant regression slope represented our measure of history bias. At the group level, a one-sample *t* test (vs. 0) was performed on the resulting regression slopes to examine whether they were statistically different from zero (i.e., whether they showed a systematic directionality across participants). This was done separately for trial lags of 1, 2, and 3 (Figure 2D). This analysis was also performed separately for trials in which the previous perceptual choice (i.e., “left” or “right”) was “repeated” versus trials in which the perceptual choice was “alternated” (Figures 5A, 5B). A paired-samples *t* test was used to compare the regression slopes between “repetition” and “alternation” trials.

### Quantifying choice history biases and estimating population prevalence using mutual information (MI)

Mutual Information (MI) is a measure of statistical dependence between two random variables that places no assumption on the form of the dependence. For two discrete variables *X* and *Y* that are distributed according to a joint probability distribution *P(X,Y)* the MI is defined as:

IX;Y=∑x,yPx,ylog2Px,yPx,yPxPyPxPy

When the probability distributions are estimated from observed data the resulting MI estimate suffers from a limited sampling bias, which causes the expectation of the estimate to be systematically larger than the true value. We correct this by subtracting the Miller-Madow bias estimate (Miller, 1955), which is given by (|X|-1)(|Y|-1)2N trl ln2, where |*X*|,  and |*Y*| are, respectively, the number of discrete values taken by the variables *X* and *Y*, and  *N*_trl_ is the number of trials used for the calculation. Statistical inference was performed via permutation testing. The relationship between *X* and *Y* was shuffled, and the resulting MI values were stored. This was repeated 1000 times (separately for each participant). The ninety-fifth percentile of the resulting permutation value was used as the threshold for inference on the MI value obtained from the unshuffled data.

We calculated the following MI values (Figure 2E): I (Orient; Resp), I (Resp-1; Resp), I (Orient; Conf), I (Conf-1; Conf). In these calculations, the number of bins for the orientation is reduced by considering neighboring levels of evidence together (e.g., seven discrete bins corresponding to the following presented angles: [−18° to −15°] [−12° to −9°] [−6° to −3°] [0°] [3°–6°] [9°–12°] [15°–18°]). Perceptual response is always represented with two discrete values (left or right). Confidence was represented with three or four discrete values (some participants never used one of the four confidence response values). For the choice history calculations, the variable Resp-1/Conf-1 is given by all trials excluding the last, the variable Resp/Conf is formed from all trials excluding the first.

### Modelling perceptual and metacognitive sensitivity and bias

Computational models of perceptual decision-making and confidence judgements, grounded largely in statistical decision theory and SDT, have successfully accounted for a range of confidence related empirical data (Kepecs et al., 2008; Sanders et al., 2016; Pouget et al., 2016; Pleskac & Busemeyer, 2010). Here, we modeled perceptual decisions and confidence ratings within an extended SDT framework (Maniscalco & Lau, 2012). This model assumes that, during yes/no detection or 2-AFC discrimination tasks, binary decisions are made by the comparison of internal evidence (indexed by a noisy decision variable [*dv*]) with a decision criterion (*c*). Across trials, evidence generated by each stimulus class (i.e., noise/signal, choice A/choice B) is sampled from a stimulus-specific normal distribution. The relative separation between the distributions (in standard deviation units) indexes the overall level of evidence available for the decisions (*dʹ*) and, hence, how well the observer can discriminate between noise and signal or between choice A and choice B. On a given trial, the probability that the choice is correct is indexed by the absolute distance between *dv* and *c* (in an unbiased observer), and, hence, statistically optimal confidence judgements should reflect this computation (Sanders et al., 2016; Pouget et al., 2016). When a discrete confidence rating scale is used, the rating on a given trial is defined by where the *dv* falls with respect to the so-called “type-2” criteria (*c2*). The *c2* are response conditional, with separate criteria for the 2 possible choices (i.e., noise/signal, choice A/choice B). Overall, there are (k-1) × 2 *c2*, where k equals the number of confidence ratings available. Figure 1B presents the model schematically for three differing levels of decision evidence: no evidence (left panel), weak evidence (middle panel), and strong evidence (right panel). The distributions and predicted effects in Figure 1B–E were produced using code developed by Urai et al., (2017) (https://github.com/anne-urai/pupilUncertainty). The x-axis ranges from −15 to 15 in these examples, and *dʹ* was set to 0.1 (no evidence), 1.58 (weak evidence), and 3.17 (strong evidence), whereas *c* was always set to 0 (unbiased observer). The flanking *c2* were set at ±3 (conservative) and ±6 (liberal) for each. To formalize the predicted relationships between evidence strength, accuracy, and confidence (Figure 1E), we simulated a normal distribution of *dv* for one response (i.e., *µ* > 0) at each level of evidence strength. All samples from the simulated distribution were split into correct and error “choices” based on their position relative to *c.* For each combination of evidence strength and choice, the level of confidence is

$$\begin{array}{c} \text{Confidence}=\frac{1}{n}\;\times \sum_{i=1}^{n}f\left(\left|dv_{i}\right|-c|\right) \end{array}$$

where *f* is the cumulative distribution function of the normal distribution

$$f\left(x\right)=\;\frac{1}{2}\;\left[1+erf\left(\frac{x}{\sigma \sqrt{2}}\right)\right]$$

which transforms the distance between *dv* and *c* into the probability of a correct response (Urai et al., 2017; Lak et al., 2014). Ten million trials were simulated, and for each iteration a binary choice was computed along with its accuracy and corresponding level of confidence. Because response times are often taken as a proxy of decision confidence (with response times increasing as a function of decreasing confidence) (Urai et al., 2017; Sanders et al., 2016) the response time prediction (Figure 1E) represents an inversion of the confidence prediction (Figure 1D).

To quantify both type-1 and type-2 performance parameters (i.e., sensitivity and bias) across different levels of evidence strength (absolute Gabor orientations) in the real data, we adopted the *meta-dʹ* approach (see Maniscalco & Lau, 2012, Fleming 2017 and Sherman et al., 2018 for extended description and discussion) as implemented using single-subject Bayesian model fits within the “HMeta-d” toolbox (Fleming, 2017: https://github.com/metacoglab/HMeta-d). *Meta-dʹ* characterizes type-2 sensitivity as the value of *dʹ* that a metacognitively optimal observer, with the same type-1 response bias (c), would have required to produce the observed type-2 (confidence) data (Maniscalco & Lau, 2012). If an observer has perfect metacognitive insight (i.e., they are always high in confidence when correct and low in confidence when incorrect) then *dʹ* will be equal to *meta-dʹ*. Importantly, because *meta-dʹ* is expressed in the same units as *dʹ*, the two can be compared directly to quantify the level of metacognitive efficiency. If the metacognitive efficiency score (*meta-dʹ* − *dʹ*) ≠ 0, then the type-2 responses (confidence ratings) are either more (positive value) or less (negative value) sensitive to the task-related evidence than the type-1 perceptual responses. We note that (*meta-dʹ/dʹ*) is often used to quantify metacognitive efficiency as a ratio of type-1 performance (Morales et al., 2018) and so we replicated our correlation analyses involving (*meta-dʹ* − *dʹ*) using (*meta-dʹ/dʹ*) (see Supplementary Figure S5). The same pattern of results was found. The metacognitive criteria (*meta-cʹ*) represent type-2 bias (*c2*) calculated within the meta-dʹ framework: the tendency to give high or low confidence ratings regardless of evidence strength. We calculated the absolute distance between *meta-cʹ* and type-1 *cʹ* (|*meta-c* *−* *c’*|) to isolate the metacognitive response bias from the perceptual response bias (Sherman et al., 2018). Lower values of |*meta-c’* *−* *c’*| indicate an overall response bias in favor of higher confidence ratings. As mentioned, *meta-c’ (c2)* values are calculated separately for each of the possible perceptual responses (i.e., “left” or “right” orientation judgements in the current study) and for each of N-1 confidence ratings available to choose from (4 in the current experiment). To streamline the analysis, we averaged over the 3 |*meta-c’* *−* *c’*| values for each response (“left” or “right”) separately to gain a single estimate of overall metacognitive response bias.

### Statistical analyses on SDT parameters

**Figure 3.**
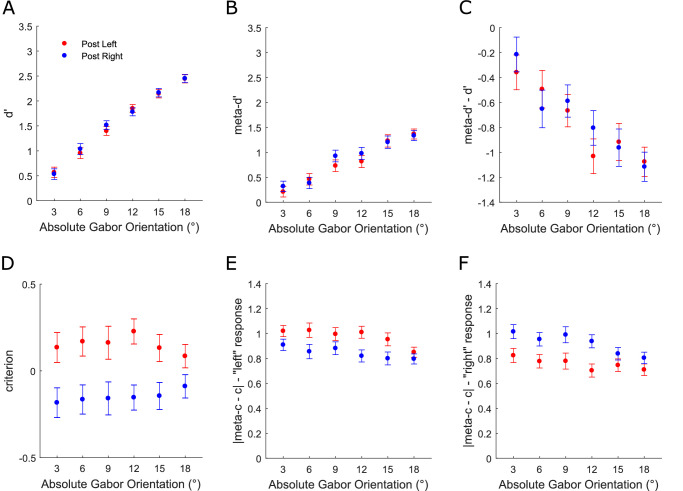
Modeling the influence of perceptual decisions on subsequent perceptual and metacognitive performance (see Methods for details). **(A)** Group-averaged *dʹ* as a function of absolute Gabor orientation and perceptual choice on the previous trial. **(B)** Group-averaged *meta-dʹ* as a function of absolute Gabor orientation and perceptual choice on the previous trial. **(C)** Group-averaged *meta-dʹ* *−* *dʹ* as a function of absolute Gabor orientation and perceptual choice on the previous trial. **(D)** Group-averaged *c* as a function of absolute Gabor orientation and perceptual choice on the previous trial. **(E)** Group-averaged |*meta-c* *−* *c*| for “left” responses as a function of absolute Gabor orientation and perceptual choice on the previous trial. **(F)** Group-averaged |*meta-c* *−* *c*| for “right” responses as a function of absolute Gabor orientation and perceptual choice on the previous trial. Note that data are not presented for the 0° orientation because meta-dʹ modeling cannot be applied when there is no veridical response. All error bars represent within-subject ± standard error (SEM).

**Figure 4.**
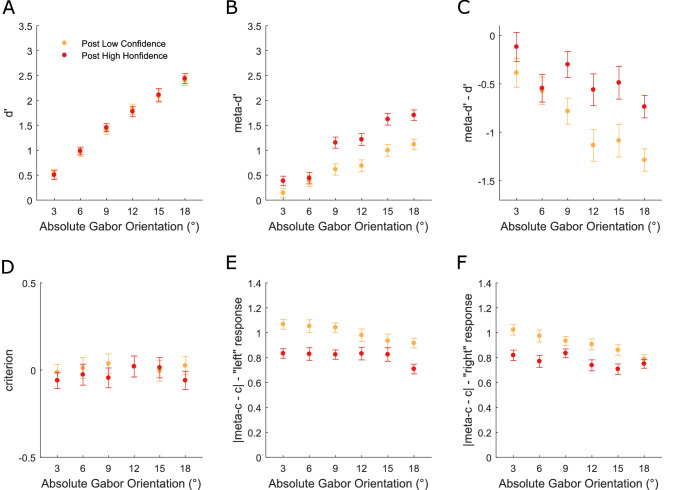
Modelling the influence of metacognitive decisions (confidence ratings) on subsequent perceptual and metacognitive performance. **(A)** Group-averaged *dʹ* as a function of absolute Gabor orientation and confidence on the previous trial. **(B)** Group-averaged *meta-dʹ* as a function of absolute Gabor orientation and confidence on the previous trial. **(C)** Group-averaged *meta-dʹ* − *dʹ* as a function of absolute Gabor orientation and confidence on the previous trial. **(D)** Group-averaged *c* as a function of absolute Gabor orientation and confidence on the previous trial. **(E)** Group-averaged |*meta-c* *−* *c*| for “left” responses as a function of absolute Gabor orientation and confidence on the previous trial. **(F)** Group-averaged |*meta-c* *−* *c*| for “right” responses as a function of absolute Gabor orientation and confidence on the previous trial. Note that data are not presented for the 0° orientation because meta-dʹ modeling cannot be applied when there is no veridical response. All error bars represent within-subject ± standard error (SEM).

**Figure 5.**
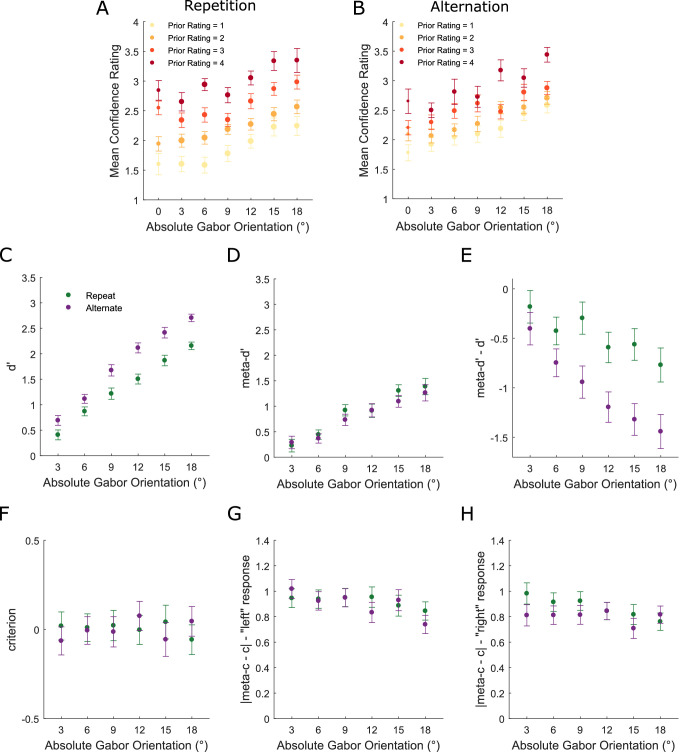
Choice history bias in confidence ratings as a function of perceptual choice hysteresis. **(A)** Group-averaged confidence ratings as a function of absolute Gabor orientation and rating on the previous trial for perceptual choice repetition trials. The size of the dots indexes the relative number of trials contributing to the group average as this was not uniform across orientations and previous ratings. **(B)** Group-averaged confidence ratings as a function of absolute Gabor orientation and rating on the previous trial for perceptual choice alternation trials. **(C)** Group-averaged *dʹ* as a function of absolute Gabor orientation and perceptual choice relative to previous choice. **(D)** Group-averaged *meta-dʹ* as a function of absolute Gabor orientation and perceptual choice relative to previous choice. **(E)** Group-averaged *meta-dʹ* *−* *dʹ* as a function of absolute Gabor orientation and perceptual choice relative to previous choice. **(F)** Group-averaged *c* as a function of absolute Gabor orientation and perceptual choice relative to previous choice. **(G)** Group-averaged |*meta-c* *−* *c*| for “left” responses as a function of absolute Gabor orientation perceptual choice relative to previous choice. **(H)** Group-averaged |*meta-c* *−* *c*| for “right” responses as a function of absolute Gabor orientation and perceptual choice relative to previous choice. Note that data are not presented for the 0° orientation because meta-dʹ modeling cannot be applied when there is no veridical response. All error bars represent within-subject ± standard error (SEM).

We compared overall perceptual sensitivity (*dʹ*) to metacognitive sensitivity (*meta-dʹ*) across all levels of evidence strength using a 2 (sensitivity measure: *dʹ, meta-dʹ*) × 6 (absolute Gabor orientation: 3°, 6°, 9°, 12°, 15°, 18°) repeated-measures ANOVA. To assess the extent to which the type-1 and type-2 SDT performance parameters were influenced by both perceptual and metacognitive choice history, trials were binned in three different ways (1. “post left”/“post right” choice trials (Figure 3); 2. “post high”/“post low” confidence trials (Figure 4); 3. “repetition”/“alternation” trials (Figure 5)) and the parameters (*dʹ, meta-dʹ, meta-dʹ* − *dʹ, c, |meta-cʹ* *−* *cʹ|:* *“left”* *responses, |meta-cʹ* *−* *cʹ|:* *“right”* *responses*) were calculated for both bins separately at each of the six levels of evidence strength. Note that the SDT parameter analyses were only performed for trial lags of −1, therefore ensuring that trials were only ever binned according to behavior on the immediately preceding trial. Additionally, the trial binning (i.e., according to high versus low confidence on the previous trial) was performed based on the data across all trials of the experiment within each participant prior to separate analyses of meta-dʹ parameters at each level of absolute Gabor orientation. This ensured that high- versus low-confidence trials were defined according to the participant's behavior across the full experiment rather than a reduced subset of trials. Repeated measures ANOVAs (2 [choice history bin] × 6 [absolute Gabor orientation: 3°, 6°, 9°, 12°, 15°, 18°]) were performed separately for each parameter. Significant interaction terms were followed up using paired samples *t* tests of the difference between the choice history bins separately at each level of evidence strength. To split the trials into relatively equal “post high” and “post low” confidence bins across all trials within each participant, the number of trials immediately following each of the 4 confidence ratings (i.e., post “1”, “2”, “3”, “4” ratings) was calculated and bins were assigned that minimized the difference in trial number between the high and low bins (median difference between bins = 69 trials [min = 7, max = 251]). This led to 10 participants having “low” bin = “1”, “2”, and “3” ratings, “high” bin = “4” ratings, 14 participants (“low” bin = “1” and “2” ratings, “high” bin = “3” and “4” ratings) and 13 participants (“low” bin = “1” ratings, “high” bin = “2”, “3”, and “4” ratings). Note that four participants were excluded from the analysis of the influence of previous confidence level on perceptual choice history bias (Supplementary Figure S1) because they had PF slope values over 3 median absolute deviations from the overall group median in at least one of the conditions here. This was due to biased perceptual or confidence decisions leading to a small number of trials being available for PF fitting after binning for these participants.

For all *t* tests and correlations (see below), we calculated the *BF*_10_ obtained from paired-samples Bayesian *t* tests (Rouder et al., 2009) or correlation hypothesis tests (Wetzels & Wagenmakers, 2012), with a prior following a Cauchy distribution and a scale factor of 0.707. *BF*_10_ quantifies the evidence in favor of the null or alternative hypotheses, where BF_10_ below 1/3 indicates evidence for the null hypothesis, above 3 indicates evidence for the alternative hypothesis and between 1/3 and 3 indicates that the evidence is inconclusive (potentially because of a lack of statistical power) (Rouder et al., 2009).

### Between-subject correlations

Both Pearson and Spearman correlation coefficients were calculated for each of the between-subject correlations of interest. Only Pearson's *r* values are shown in the corresponding figures.

## Results

### Overall performance exhibited signatures predicted by computational models of decision-making and metacognition

Thirty-seven human observers performed a two-alternative forced choice (2-AFC) orientation discrimination task (Figure 1A). Participants judged whether a briefly presented Gabor patch was tilted leftward or rightward of the vertical plane and reported the level of confidence they felt in their decision (on a scale of 1 – “Not confident at all” to 4 – “Highly confident”). The true orientation (and, hence, task difficulty) was manipulated from trial to trial. This design allowed us to test predictions arising from a recently proposed computational model of perceptual decision-making and metacognition based on Bayesian statistical confidence and signal detection theory (SDT), as defined in Figure 1B (and Methods). Briefly, human decisions have been modeled as the comparison of an internal decision variable (DV), representing the evidence in favor of one or other choice in 2-AFC tasks, against a decision criterion (C). Under this model, confidence in the decision is given by the distance of the DV from C (Galvin et al., 2003; Maniscalco & Lau, 2012; Urai et al., 2017; Kepecs et al., 2008; Sanders et al., 2016; Hebart et al., 2016; Masset et al., 2020). When a discrete confidence rating scale is used, the level of confidence is defined by where the DV falls with respect to the so-called type-2 criteria (c_1_, c_2_, … c_N−1)_), where N indexes the number of possible ratings. A confidence rating of k will follow if the DV falls in the interval (c_k−1_, c_k_).

This model gives rise to several predictions regarding the relationships between stimulus evidence, accuracy and decision confidence (Urai et al., 2017; Sanders et al., 2016; Hangya, Sanders, & Kepecs, 2016; Drugowitsch, 2016; Fleming & Daw, 2017; although see Adler & Ma, 2018 for criticism of this model): (1) Accuracy should scale with evidence strength (Figure 1C); (2) Conditioning type-1 performance on high or low confidence ratings should change the slope of the relationship between stimulus evidence and accuracy, with a steeper slope for high relative to low confidence trials (Figure 1C); (3) Confidence should increase with evidence strength for correct trials, but decrease with evidence strength for incorrect trials (Figure 1D); (4) Even when there is no veridical evidence in favor of one response or other, confidence should be above 0 (Figure 1D). These predictions were all confirmed in our data. Accuracy increased as a function of evidence strength, but the slope of the stimulus evidence-accuracy relationship was steeper for high- relative to low-confidence trials (Figure 1F). Confidence increased with evidence strength for correct trials but decreased with evidence strength for incorrect trials (Figure 1G). Accordingly, response time decreased as a function of evidence strength for correct trials but increased for incorrect trials (Figure 1H). Finally, participants reliably reported some level of confidence in decisions even when the Gabor patch was vertically aligned, and, hence, there was no informative evidence (Figure 1G).

### Choice history bias occurs in both perceptual and metacognitive decisions but is stronger in metacognition

Next, we investigated the degree to which choice history biases both perceptual and metacognitive responses. Across all trials, no systematic group-level bias in favor of either choice was apparent (*t* test of psychometric function [PF] thresholds versus 0°: *t*(36) = 0.1497, *p* = 0.8818, Bayes factor (*BF*_10_) = 0.179) (Figure 2A). However, in line with previous studies (Urai et al., 2017; Braun et al., 2018; Bonaiuto et al., 2016; Abrahamyan et al., 2016; Urai et al., 2019; Fernberger, 1920), group-averaged PFs conditioned on the previous response were shifted toward the previous response (“left”/“right” responses were more likely after “left”/“right” responses, respectively) despite randomly-ordered presentations (Figure 2A). Post-left PF thresholds were significantly biased away from veridical 0° (*t*(36) = 3.1295, *p* = 0.0035, *BF*_10_ = 10.462), as were post-right PF thresholds, but in the opposite direction (*t*(36) = −2.5466, *p* = 0.0153, *BF*_10_ = 2.9235). Accordingly, post-left thresholds were significantly different from post-right thresholds (*t*(36) = 4.2498, *p* < 0.001, *BF*_10_ = 177.4). The effect remained significant for trial lags of two (t(36) = 5.9966, *p* < 0.001, *BF*_10_ = 2.3930e + 04) and three (*t*(36) = 5.91, *p* < 0.001, *BF*_10_ = 1.8667e + 04) (Figure 2B) but was no longer significant for trial lag four (*t*(36) = 1.7667, *p* = 0.086, *BF*_10_ = 0.7217). It has been suggested that confidence on a given trial modulates the likelihood of the perceptual choice being subsequently repeated (Urai et al., 2017; Samaha et al., 2019; Bosch et al., 2020). However, we did not find any influence of preceding confidence on perceptual history bias, with the bias occurring when confidence was both low and high on the previous trial (Supplementary Figure S1).

Next, we investigated the degree of metacognitive history bias. Confidence increased as a function of absolute orientation (i.e., sensory evidence) but, in line with previous research (Rahnev et al., 2015; Mei et al., 2023), was also shifted toward previous trial ratings (i.e., “high”/“low” were more likely after “high”/“low” ratings, respectively) (Figure 2C). A regression analysis confirmed that confidence was positively predicted by ratings on the previous trial across participants (*t* test of slopes versus 0: *t*(36) = 11.7028, *p* < 0.001, *BF*_10_ = 9.3215e + 10) (Figure 2D). The effect remained statistically significant for all trial lags up to 25 (all *p*s < 0.05), thereby considerably outlasting the perceptual bias. Note that both the perceptual and confidence history biases were also present when we restricted the analyses to trials after correct responses only (see Supplementary Figure S2) and, hence, were independent of potential error awareness mechanisms.

To calculate within-participant significance and estimate population prevalence of the observed biases, we performed additional analyses using mutual information (MI) (Ince et al., 2017; Ince, Paton, Kay, & Schyns, 2021). MI provides an assumption free measure of dependence with effect sizes on a common meaningful scale (bits) across variables with different characteristics (i.e., different dimensionality and/or number of samples). Hence, to our knowledge for the first time, we could quantify and compare how strongly both perceptual and metacognitive responses of each participant were related to the objective evidence at hand versus recent choices. First, we quantified the strength of dependence between stimulus evidence (orientation of the Gabor [Orient]) and both perceptual responses and confidence ratings (Figure 2E). We then quantified, on the same scale, the choice history biases in both confidence ratings and perceptual responses (see Method for details). Supplementary Figure S3 highlights how the MI measures relate to the model-based bias measures displayed in Figures 1B and Figure 1C.

As expected, the highest dependence was found between objective evidence (Orient) and perceptual responses (Resp) (Figure 2E). Interestingly, this was stronger than the dependence between objective evidence and confidence ratings (*t*(36) = 11.6448, *p* < 0.001, *BF*_10_ = 8.1307e + 10), suggesting suboptimal metacognitive performance. The confidence history bias was stronger than the perceptual history bias (*t*(36) = 6.25, *p* < 0.001, *BF*_10_ = 4.9486e + 04) and in fact had roughly the same influence on confidence as current trial evidence (*t*(36) = 0.384, *p* = 0.7032, *BF*_10_ = 0.1894). Statistical inference was performed nonparametrically within individual participants based on 1000 permutations of the data. In our sample, 13/37 participants showed significant perceptual history bias (at *p* = 0.05). Therefore the population prevalence (Ince et al., 2021; Donhauser, Florin, & Baillet, 2018; Allefeld, Görgen, & Haynes, 2016) of perceptual history bias detectable in our experiment is 31.7% (14.6%-48.8%; maximum likelihood estimate with 95% bootstrap confidence interval). The majority of those showing significant perceptual history bias tended to repeat their previous responses (N = 10), with only three tending to alternate (Urai et al., 2017; Abrahamyan et al., 2016; Urai et al., 2019). Across participants, perceptual history bias was inversely related to the effect of evidence on perceptual responses within trials (Figure 2F: Pearson's *r* = −0.55, *p* < 0.001, *BF*_10_ = 64.297). However, the influence of evidence was stronger in most participants (MI [Orient; Resp] > MI [Resp-1; Resp], 35/37 participants) than the influence of choice history (MI [Resp-1; Resp] > MI (Orient; Resp], 2/37). Thirty-four of 37 participants showed significant metacognitive history bias (at *p* = 0.05), which implies a population prevalence of 91.4% (80.1%–100%). All participants showing significant metacognitive history bias tended to repeat previous confidence ratings. Across participants metacognitive history bias was inversely related to the effect of evidence on confidence within trials (Figure 2G: Pearson's *r* = −0.53, *p* < 0.001, *BF*_10_ = 29.059), with relatively even subgroups of participants for whom current evidence dominated confidence judgements (MI [Orient; Conf] > MI [Conf-1; Conf], 16/37) versus those for whom rating history dominated judgements (MI [Conf-1; Conf] > MI [Orient; Conf], 21/37).

### Uncovering the influence of choice history on perceptual and metacognitive decisions with computational behavioral modeling

To explore the relationship between perceptual and metacognitive choice history biases, we returned to the decision-making model (defined in Methods and Figure 1B) to formally test which aspects of both perceptual (type-1) and metacognitive (type-2) performance were affected by previous choices. Type-1 performance encompasses traditional measures of perceptual sensitivity (*dʹ*) and bias (*c*), whereas type-2 performance encompasses measures of metacognitive sensitivity (*meta-dʹ*) and bias (*meta-c*) (Maniscalco & Lau, 2012; Fleming, 2017). *Meta-dʹ* represents the type-1 *dʹ* value expected to give rise to the observed confidence data under the assumption that the observer has perfect metacognitive sensitivity (i.e., *dʹ* = *meta-dʹ* when confidence is always high when correct and low when incorrect). To quantify metacognitive efficiency (or in other words how much of the information present in the type-1 performance participants make use of in their type-2 decisions), we can subtract *dʹ* from *meta-dʹ*. If *meta-dʹ* − *dʹ* ≠ 0, then confidence ratings are either more (positive) or less (negative) sensitive to the task-related evidence than the perceptual responses. The metacognitive criteria (*Meta-c*) index the tendency to give high/low confidence ratings regardless of evidence (metacognitive response bias). Their absolute distance from type-1 *c* (|*meta-c* − *c*|) represents the level of evidence needed to increase confidence ratings from low to high (Sherman, Seth, & Barrett, 2018). Unlike type-1 *c*, *meta-c* values are calculated separately for each possible perceptual response (“left”/“right” orientation judgements). Additionally, there are N-1 *meta-c* for each response, where N indexes the number of possible ratings (four in the current experiment). To simplify the analysis, we averaged over the 3 |*meta-c* − *c*| values for each response (“left”/“right”) separately (see Methods).

First, we assessed whether overall metacognitive sensitivity (*meta-dʹ*) systematically deviated from perceptual sensitivity (*dʹ*). Across orientations, confidence judgements were less reflective of the evidence than perceptual judgements, with mean *meta-dʹ* being lower than mean *dʹ* (compare Figures 3A and 3B). A repeated-measures analysis of variance (ANOVA: 2 [sensitivity measure: *dʹ*, *meta-dʹ*] × 6 [absolute orientation {evidence}: 3°, 6°, 9°, 12°, 15°, 18°]) revealed that *meta-dʹ* was significantly lower than *dʹ* (main effect: *F*(1, 36) = 58.818, *p* < 0.001), and the difference increased as a function of orientation (Figure 3C) (interaction: *F*(5, 180) = 13.614, *p* < 0.001). Hence, participants were generally unable to make use of all information available for perceptual judgements when estimating their confidence (suboptimal metacognition (Shekhar & Rahnev, 2021)), in line with the MI results. To investigate the influence of previous perceptual choices, we calculated the type-1 and type-2 model parameters separately for “post-left” and “post-right” decision trials across each level of evidence strength. We then performed repeated-measures ANOVAs (2 [previous choice: “left”/“right”] × 6 [absolute orientation: 3°, 6°, 9°, 12°, 15°, 18°]) for each parameter.

Previous perceptual choice did not influence either perceptual or metacognitive sensitivity (Figures 3A–C), neither *dʹ*, *meta-dʹ* nor *meta-dʹ* − *dʹ* (*F* values ≤ 1.086, *p* values ≥ 0.37). However, type-1 *c* was biased toward the previous perceptual choice across all orientations (Figure 3D: main effect: *F*(1, 36) = 20.344, *p* < 0.001; interaction: *F*(5, 180) = 1.619, *p* = 0.157), in line with the PF analysis. Metacognitive criteria (|*meta-c* − *c*|) were biased in a response-dependent manner (Figures 3E, 3F). When participants responded “left,” they displayed higher meta-criteria when they had also responded “left” on the previous trial (repetition) compared to when they had responded “right” (alternation) (main effect: *F*(1, 36) = 12.983, *p* < 0.001; interaction: *F*(5, 180) = 1.603, *p* = 0.162). Accordingly, when participants responded “right,” they displayed higher meta-criteria when they had responded “right” on the previous trial (repetition) compared to when they had responded “left” (alternation) (main effect: *F*(1, 36) = 14.52, *p* < 0.001; interaction: *F*(5, 180) = 2.427, *p* = 0.037). The interaction term in the “right” response analysis was driven by the effect not being significant for the two largest orientations. The effect was significant for 3°, 6°, 9°, 12° (*t* values ≥ 3.164, *p* values ≤ 0.003, *BF*_10_ ≥ 11.352) but not 15° (*t*(36) = 1.775, *p* = 0.084, *BF*_10_ = 0.731) nor 6° (*t*(36) = 1.972, *p* = 0.056, *BF*_10_ = 1). In sum, perceptual choices influenced decision criteria for both perceptual and metacognitive subsequent choices.

To investigate the influence of the previous metacognitive choice, we performed the same analysis but this time for “post-high” and “post-low” confidence trials (two bins split as evenly as possible within each participant: see Methods). Previous confidence did not influence perceptual sensitivity (Figure 4A: *dʹ* main effect: *F*(1, 36) = 0.076, *p* = 0.784; interaction: *F*(5, 180) = 0.162, *p* = 0.976), but it did influence subsequent metacognitive sensitivity (Fig. 4B: *meta-dʹ* (main effect: *F*(1, 36) = 48.972, *p* < 0.001; interaction: *F*(5, 180) = 4.617, *p* = 0.001)) and metacognitive efficiency (Fig. 4C: *meta-dʹ* *−* *dʹ* main effect: *F*(1, 36) = 33.194, *p* < 0.001; interaction: *F*(5, 180) = 2.375, *p* = 0.041). The interaction terms in both the metacognitive sensitivity (*meta-dʹ*) and efficiency (*meta-dʹ* − *dʹ*) analyses were driven by the effect increasing as a function of orientation (Figures 4B, 4C). For metacognitive sensitivity, follow-up *t* tests showed that the effect was significant for orientations of 3°, 9°, 12°, 15°, 18° (*t* values ≥ 2.413, *p* values ≤ 0.021, *BF*_10_ ≥ 2.239) but not for 6° (*t*(36) = 0.457, *p* = 0.65, *BF*_10_ = 0.195). For metacognitive efficiency, the effect was significant for 9°, 12°, 15°, 18° (*t* values ≥ 3.368, *p* values ≤ 0.002, *BF*_10_ ≥ 18.449) but not for 3° (*t*(36) = 1.737, *p* = 0.091, *BF*_10_ = 0.689) nor 6° (*t*(36) = 0.18, *p* = 0.858, *BF*_10_ = 0.179).

In contrast to the perceptual history bias, type-1 *c* was not influenced by confidence on the previous trial (Figure 4D: main effect: *F*(1, 36) = 1.419, *p* = 0.241; interaction: *F*(5, 180) = 0.645, *p* = 0.666). However, |*meta-c* − *c*| were significantly reduced after “high” relative to “low” confidence responses, both for “left” (Figure 4E) (main effect: *F*(1, 36) = 43.086, *p* < 0.001; interaction: *F*(5, 180) = 1.481, *p* = 0.198) and “right” responses (Figure 4F) (main effect: *F*(1, 36) = 31.366, *p* < 0.001; interaction: *F*(5, 180) = 3.025, *p* = 0.012), indicating that “high”/“low” confidence ratings were more likely following “high”/“low” ratings, respectively. The interaction term in the “right” response analysis was driven by the previous rating effect decreasing as a function of orientation (Figure 4F: linear contrast *F*(1, 36) = 6.771, *p* = 0.013). Follow-up *t* tests showed that the effect was significant for orientations of 3°, 6°, 9°, 12°, 15° (*t* values ≥ 2.739, *p* values ≤ 0.01, *BF*_10_ ≥ 4.37) but not for 18° (*t*(36) = 1.066, *p* = 0.203, *BF*_10_ = 0.299). Hence, we show for the first time that metacognitive choice history influences all aspects of metacognitive performance (sensitivity, efficiency, and bias) but does not influence perceptual sensitivity nor bias.

### Choice alternation is associated with increased perceptual sensitivity but reduced metacognitive efficiency

Next, we investigated directly whether repeating (versus alternating) the previous choice was associated with changes in either perceptual or metacognitive performance. Figure 5 plots metacognitive history bias effects separately for “repetition” (Figure 5A) and “alternation” trials (Figure 5B). For both, confidence increased as a function of orientation but also tended to be shifted toward previous ratings. Confidence was positively predicted by previous ratings for both repetition (*t*(36) = 11.88, *p* < 0.001, *BF*_10_ = 1.4145e + 11) and alternation trials (*t*(36) = 6.6953, *p* < 0.001, *BF*_10_ = 1.7697e + 05). However, the effect was significantly stronger for repetition trials (*t*(36) = 5.0343, *p* < 0.001, *BF*_10_ = 1.5439e + 03). Intriguingly, computational modeling revealed a novel dissociation of perceptual and metacognitive sensitivity induced by disengagement from choice hysteresis. When participants alternated from their previous choice, they were more likely to be correct than when they repeated (Figure 5C: *dʹ* main effect: *F*(1, 36) = 68.841, *p* < 0.001; interaction: *F*(5, 180) = 2.763, *p* = 0.02). The effect was significant at all orientations (*t* values ≥ 2.709, *p* values ≤ 0.01, *BF*_10_ ≥ 4.1) but increased as a function of orientation (linear contrast: *F*(1, 36) = 13.284, *p* = 0.001). However, this improvement in perceptual sensitivity for alternation trials was not reflected in metacognitive sensitivity (Figure 5D: *meta-dʹ* (main effect: *F*(1, 36) = 1.311, *p* = 0.26; interaction: *F*(5, 180) = 0.932, *p* = 0.462)). Hence, objective accuracy increased for alternation relative to repetition trials whereas metacognitive efficiency decreased (Figure 5E: *meta-dʹ* − *dʹ* main effect: *F*(1, 36) = 27.262, *p* < 0.001; interaction: *F*(5, 180) = 2.321, *p* = 0.045). The *meta-dʹ* *−* *dʹ* effect was significant at orientations of 6°, 9°, 12°, 15°, 18° (*t* values ≤ −2.2, *p* values ≤ 0.033, *BF*_10_ ≥ 1.552), but not 3° (*t*(36) = −1.303, *p* = 0.201, *BF*_10_ = 0.385), and increased as a function of orientation (*F*(1, 36) = 9.899, *p* = 0.003).

Choice hysteresis did not influence either perceptual or metacognitive decision criteria (Figures 5F–H: *F* values ≤ 1.685, *p* values ≥ 0.14). Overall, participants lacked full insight into the increased likelihood of being correct when they alternated from their previous perceptual response.

### Choice history biases are associated with reduced perceptual and metacognitive sensitivity, but not reduced metacognitive efficiency, across participants

**Figure 6.**
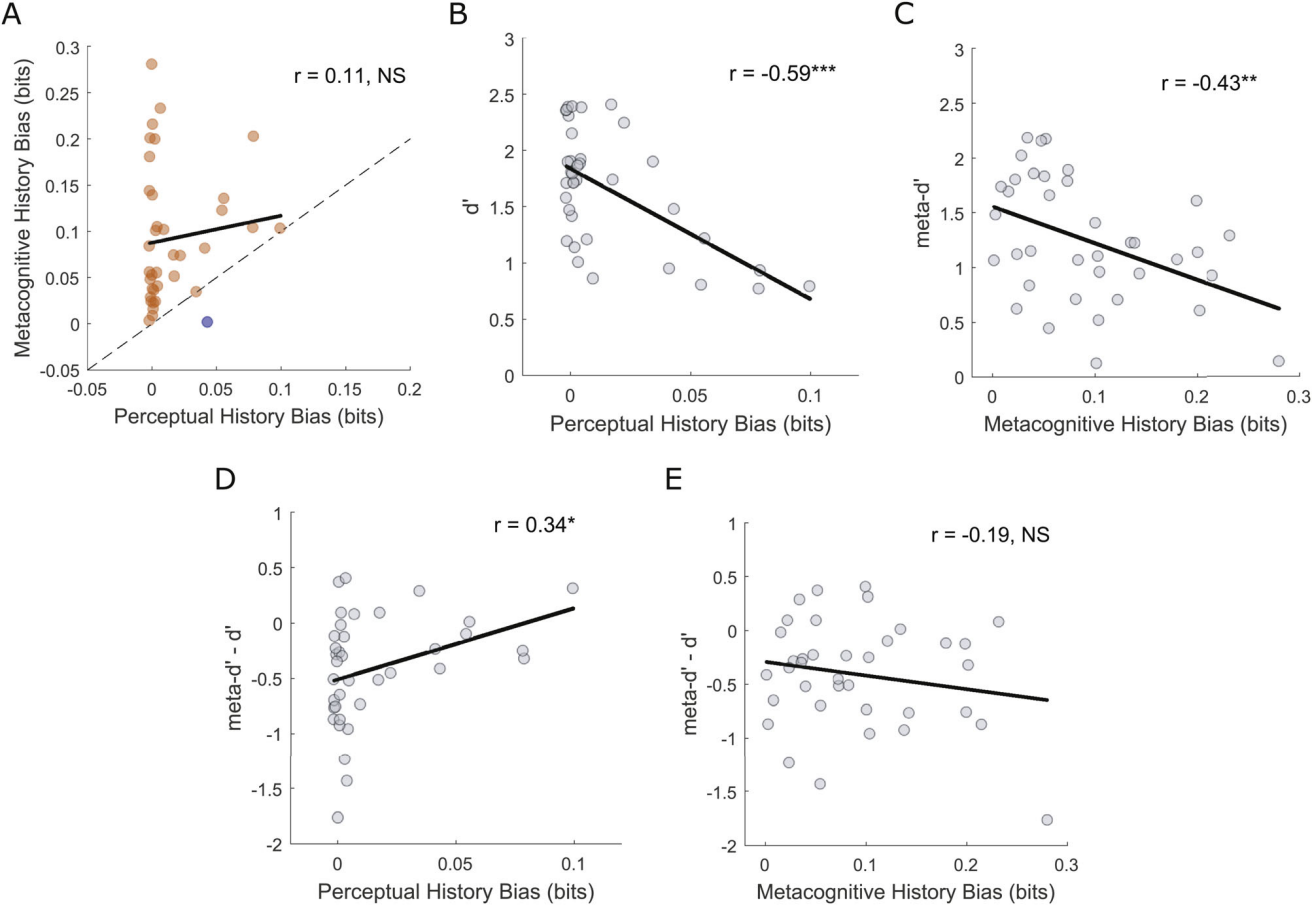
Between-subject Pearson correlations. **(A)** Relationship between perceptual and metacognitive choice history biases. Metacognitive history bias was stronger in most participants (MI (Conf-1; Conf) > MI(Resp-1; Resp), 36/37 participants) than perceptual history bias (MI (Resp-1; Resp) > MI (Conf-1; Conf), 1/37 participants). **(B)** Relationship between perceptual choice history bias and perceptual sensitivity (*dʹ*)*.* **(C)** Relationship between metacognitive choice history bias and metacognitive sensitivity (*meta-dʹ*). **(D)** Relationship between perceptual choice history bias and metacognitive efficiency (*meta-dʹ* *−* *dʹ*). **(E)** Relationship between metacognitive choice history bias and metacognitive efficiency (*meta-dʹ* *−* *dʹ*). Solid black lines represent least-squares regression slopes. ***p < 0.001, **p < 0.01, *p < 0.05, NS *p* > 0.05.

Finally, we investigated the correlation between perceptual and metacognitive history biases (Figure 6A) and whether they contribute to suboptimal perceptual and metacognitive sensitivity, across participants. The strength of perceptual bias did not predict the strength of metacognitive bias (Pearson's *r* = 0.1072, *p* = 0.5278, *BF*_10_ = 0.156). Metacognitive history bias was stronger in most participants (MI [Conf-1; Conf] > MI[Resp-1; Resp], 36/37 participants) than perceptual history bias (MI [Resp-1; Resp] > MI [Conf-1; Conf], 1/37 participants).

History biases have previously been linked to reduced perceptual (Abrahamyan et al., 2016) and metacognitive sensitivity (Rahnev et al., 2015), and we replicated these findings here. The perceptual bias was inversely related to perceptual sensitivity (*dʹ*) (Figure 6B: *r* = −0.5877, *p* < 0.001, *BF*_10_ = 179.836), and the metacognitive bias was inversely related to metacognitive sensitivity (*meta-dʹ*) (Figure 6C: *r* = −0.4315, *p* = 0.0077, *BF*_10_ = 4.353). Perceptual history bias was not significantly associated with metacognitive sensitivity (*meta-dʹ*), and metacognitive history bias was not significantly associated with perceptual sensitivity (*dʹ*) (Supplementary Figure S4).

Previously, a negative correlation was found between metacognitive history bias and metacognitive sensitivity (as quantified by the area under the Type-2 receiver operating characteristic [ROC] curve [Type-2 AUC]) (Rahnev et al., 2015). However, neither type-2 AUC nor *meta-dʹ* account for type-1 performance and, hence, do not represent pure measures of metacognitive insight/efficiency (Maniscalco & Lau, 2012; Fleming, 2017). Therefore, to establish the relationships between perceptual and metacognitive history biases and metacognitive efficiency, we correlated both with *meta-dʹ* − *dʹ*. A weak positive correlation was found between perceptual history bias and metacognitive efficiency (Figure 6D: *r* = 0.3437, *p* = 0.0373, *BF*_10_ = 1.101). The *BF*_10_ did not provide strong evidence for the alternative hypothesis; therefore we do not interpret this further. However, a one-tailed analysis to test for a negative relationship revealed strong evidence for the null hypothesis (*BF*_10_ = 0.07). A nonsignificant negative relationship was observed between metacognitive history bias and metacognitive efficiency (Figure 6E: *r* = −0.1852, *p* = 0.2726, *BF*_10_ = 0.233). Hence, when the contribution of type-1 performance to absolute metacognitive sensitivity was factored out, history biases were not significantly associated with reduced metacognitive efficiency across participants. Note that similar results were found using a ratio measure of metacognitive efficiency (Supplementary Figure S5).

Using MI to quantify history biases eliminates information about the bias direction (i.e., “Repeater” versus “Alternator”). For the sake of completeness, the same correlation analyses using metrics which retain the bias direction are reported in Supplementary Figure S6.

## Discussion

Human decisions are often influenced by sources other than the relevant information (Wilson & Dunn, 2004; Johansson et al., 2005; Drugowitsch, Moreno-Bote, & Pouget, 2014; Rahnev & Denison, 2018). Understanding suboptimal decision-making represents a fundamental enterprise in modern psychology and neuroscience (Wyart & Koechlin, 2016). In line with previous studies, we show that choice history represents a source of task-irrelevant choice variability, both for perceptual decisions (Urai et al., 2017; Braun et al., 2018; Bonaiuto et al., 2016; Abrahamyan et al., 2016; Urai et al., 2019; Fernberger, 1920; Pascucci et al., 2019) and confidence reports (Rahnev et al., 2015). Most participants displayed positive history biases: they were more likely to repeat perceptual decisions and confidence ratings, even though stimuli were presented in a random order and, hence, previous choices were of no relevance. Crucially, we quantified both perceptual and metacognitive history biases on a common effect size scale (using MI) and estimated single-subject significance and population prevalence of the respective effects. Additionally, by using computational modeling of perceptual decisions and confidence ratings, we were able to uncover latent parameters that are influenced by choice history at different levels of the decision-making hierarchy. Across participants, perceptual and metacognitive history biases did not correlate with each other but were independently associated with reduced perceptual and metacognitive sensitivity, whereas neither bias predicted metacognitive efficiency. We show for the first time that the perceptual and metacognitive biases influence distinct type-1 (perceptual) and type-2 (metacognitive) aspects of decision-making, and the metacognitive bias is stronger, significant over longer trial lags, and likely to be more prevalent in the general population. These observations are of fundamental relevance for contemporary models of decision-making and confidence, suggesting that recent confidence represents a mental shortcut (heuristic) that informs self-reflection when more relevant information is unavailable.

A normative model posits that confidence computations reflect the probability of being correct in a statistically optimal manner (Kepecs et al., 2008; Sanders et al., 2016; Hangya et al., 2016; Pouget, Drugowitsch, & Kepecs, 2016: Masset et al., 2020), in line with suggestions that the computation of confidence arises from the same neural processes as the decision itself (Gherman & Philiastides, 2015; Kiani & Shadlen, 2009; Meyniel, Schlunegger, & Dehaene, 2015; van den Berg et al., 2016). The normative model relates confidence to the available evidence through a conditional Bayesian posterior probability (Braun et al., 2018; Hangya et al., 2016; Drugowitsch et al., 2014; although see Adler & Ma, 2018 for criticism of this model), and several of the model predictions were met in the current dataset using explicit confidence ratings of visual discrimination performance (see Figures 1B–H). This suggests that subjective confidence is to some extent consistent with normative statistical principles, although it should be noted that a first-order normative model is not the only model that gives rise to such predictions (Fleming & Daw, 2017; Adler & Ma, 2018). However, the influence of choice history on confidence ratings (see Figures 2C–G) shows that the normative model alone cannot fully account for subjective confidence. Rather, the normative computation may be one of several determinants of confidence (Sanders et al., 2016), and differential weighting of these determinants may explain individual differences in the degree of metacognitive history bias and overall metacognitive sensitivity. Other factors that have been suggested to influence confidence judgements include context (Huettel, Song, & McCarthy, 2005), social pressure (Bahrami et al., 2012; Bang et al., 2017), attention (Denison et al., 2018; Rahnev et al., 2011), and fatigue (Maniscalco, McCurdy, Odegaard, & Lau, 2017). Our approach allowed us to quantify and compare the degree to which confidence judgements were driven by objective evidence versus preceding confidence ratings. Surprisingly, we found a relatively even split of participants for whom the objective evidence most strongly influenced confidence versus participants for whom previous ratings were a stronger influence (Figure 2G). In contrast, all but one participant showed a stronger influence of objective evidence on perceptual choices than the influence of previous choices (Figure 2F). Metacognitive judgements are thus more susceptible to bias from extraneous factors than perceptual decisions, an observation which may be of practical relevance in terms of learning, error monitoring and psychological well-being (van den Berg et al., 2016; Desender et al., 2018; Yeung & Summerfield, 2012; Bahrami et al., 2012; Folke et al., 2016; Rouault et al., 2018; Benwell et al., 2022). Further research may investigate whether primarily “history-” versus “evidence-”based metacognitive styles meaningfully predict differences in influential traits such as cognitive flexibility, personality, or psychiatric symptomology.

The current results align with models positing that confidence judgements arise from processes that are to some degree dissociable from the decision process itself (Maniscalco & Lau, 2012; Fleming & Daw, 2017; Pleskac & Busemeyer, 2010), with distinct neural implementations and independent influences. Evidence supporting such a dissociation has come from neuroimaging (Fleming, Weil, Nagy, Dolan, & Rees, 2010; Peters et al., 2017; Hebart et al., 2016; Morales, Lau, & Fleming, 2018; Fleming et al., 2012; Bang & Fleming, 2018; Lebreton, Abitbol, Daunizeau, & Pessiglione, 2015; Murphy, Robertson, Harty, & O'Connell, 2015; De Martino, Fleming, Garrett, & Dolan, 2013; Benwell et al., 2017), psychophysics (Samaha et al., 2019; Zylberberg, Roelfsema, & Sigman, 2014; Ais, Zylberberg, Barttfeld, & Sigman, 2016; Shekhar & Rahnev, 2021), brain stimulation (Rounis, Maniscalco, Rothwell, Passingham, & Lau, 2010; Rahnev, Maniscalco, Luber, Lau, & Lisanby, 2012) and clinical (David et al., 2012; Fleming, Ryu, Golfinos, & Blackmon, 2014; Del Cul, Dehaene, Reyes, Bravo, & Slachevsky, 2009; Hoven et al., 2019) studies. Several aspects of our findings accord with a “second-order” computation of confidence. First, participants were generally unable to make use of all the information available for their perceptual decisions when rating confidence, which indicates “noise” in the metacognitive system and suboptimal insight (Maniscalco, Peters, & Lau, 2016; McCurdy et al., 2013). Additionally, perception and metacognitive history biases were uncorrelated across participants (Figure 6A) and impacted on distinct latent decision-making parameters (Figures 3, 4). For instance, type-2 (metacognitive) decision criteria were modulated as a function of prior confidence ratings independently of the type-1 (perceptual) criteria (Figures 4D–F) and alternating from choice hysteresis was associated with increased perceptual sensitivity but reduced metacognitive insight (Figures 5C–E). This dissociation when disengaging from choice hysteresis, reported here for the first time, adds to previous reports suggesting that accuracy and confidence can be uncoupled even in healthy participants (Rahnev et al., 2011; Rahnev et al., 2012; Maniscalco et al., 2016). Biasing of confidence judgements by factors which do not influence 1st-order choices (such as previous confidence ratings here) might partially explain why many studies have observed sub-optimal metacognitive efficiency (indexed by measures such as *meta-dʹ* − *dʹ* and *meta-dʹ/dʹ*) even in healthy participants. Thus confidence computations must operate, at least partly, on an axis that is dissociable from type-1 decisions. We did find evidence for some level of interaction between perceptual and metacognitive history biases. The metacognitive bias was strongest for trials in which the perceptual choice was repeated, though it remained significant also for alternation trials (Figures 5A, 5B). This suggests that some level of “shared” hysteresis occurs across both systems. However, in contrast to previous findings (Urai et al., 2017; Braun et al., 2018; Samaha et al., 2019; Bosch et al., 2020), preceding confidence had no influence on the likelihood of the perceptual choice being repeated. Subtle but important differences in experimental designs may explain this discrepancy (see Supplementary Figure S1).

Why might perceptual and metacognitive decision processes be dissociable? One possibility is that the nature of everyday decision-making renders the use of all type-1 information for metacognitive reflection either impossible or unnecessary (Maniscalco et al., 2016). As is known for decision-making, metacognitive judgements might rely partly on heuristics and simplifications that result in systematic biases under specific conditions including laboratory-based tasks with high levels of uncertainty (Peters et al., 2017; Maniscalco et al., 2016; Griffin & Tversky, 1992; Tversky & Kahneman, 1974; Zylberberg, Barttfeld, & Sigman, 2012). In natural settings, it may generally be advantageous to assume statistical regularity of environmental stimuli and to default to this model/heuristic under conditions of high uncertainty (Pascucci et al., 2019; Kiyonaga et al., 2017). If the metacognitive system has less access to objective evidence than the perceptual system, then stronger history biases of confidence ratings are likely to occur. Indeed, here confidence ratings were less sensitive to the objective evidence than perceptual choices and were also more strongly biased by previous ratings. Future studies should investigate whether apparent dissociation of first-order and confidence history biases is a phenomenon that can be observed to the same degree across different decision tasks (i.e., discrimination vs. detection), sensory modalities (i.e., vision, touch, audition), and cognitive domains (i.e., perception vs. memory).

The mechanisms underlying history biases remain unclear, although neural signatures encoding previous perceptual choices have been identified across various sensory, associative, and motor brain regions (John-Saaltink et al., 2016; Papadimitriou, White, & Snyder, 2017; Hwang, Dahlen, Mukundan, & Komiyama, 2017; Akaishi, Umeda, Nagase, & Sakai, 2014; Urai & Donner, 2022). Recent studies have investigated perceptual history bias within the context of computational models of decision-making. The drift-diffusion model (Ratcliff & McKoon, 2008) represents an extension of classic SDT incorporating single-trial dynamics of evidence accumulation. Under this model, biasing of the type-1 criterion by previous choices (Figure 3D) could occur because of asymmetry in either the starting point or drift rate of the evidence accumulation process. Urai et al. (2019) showed compelling evidence across six tasks that drift rate bias provides the best account, in line with persistence of decisional weights over time/trials (Bonaiuto et al., 2016; Pascucci et al., 2019), an interpretation that is in line with our results. However, it is important to acknowledge that we have not developed process models of the history biases here and that alternative mechanisms may have contributed to the observed effects. For instance, in contrast to trial-by-trial updating of decision-making parameters such as the type-1 and type-2 decision criteria, slower drifts over time may have contributed (Lak et al., 2020; Gupta & Brody, 2022). This may be particularly relevant for the metacognitive history bias, which remained significant up to a trial lag of 25. Further research should disambiguate trial-by-trial criterion updates from slow drifts over time (Gupta & Brody, 2022) and model the temporal dynamics of both type-1 and type-2 decisions (Pleskac & Busemeyer, 2010) to ascertain the mechanism(s) underlying history induced criterion shifts (Figures 4E, 4F). Additionally, by combining such an approach with functional neuroimaging (Hebart et al., 2016; Kiani & Shadlen, 2009), neural correlates of model parameters may reveal the neural implementations underlying both perceptual and metacognitive choices themselves, along with history biases.

To our knowledge, this study is the first to report estimates of the population prevalence of both perceptual and metacognitive choice history biases. We used information theoretic statistics to quantify aspects of decision-making within individual participants on a common effect size scale. These measures also enable computationally efficient nonparametric within-participant inference (Ince et al., 2021). This approach could be widely applied to different questions in studies of decision-making. We found that metacognitive history bias was significant in almost all our sample (34/37), allowing us to infer an estimate of the population prevalence of 91.5% (80.1%-100%) (maximum likelihood with 95% bootstrap confidence interval). That is, we can expect that at least 80% of the general population would have an effect detectable with our experimental design (i.e., statistically significant at *p* = 0.05 from 416 trials). The perceptual history bias was significant at the group level but was only significant in 13/37 of our sample, yielding a population prevalence estimate of 31.7% (14.6%–48.8%). Statistical inference in psychology traditionally focusses on population mean effects, but we argue that it is crucial to determine the degree to which the effects can be reliably observed within individuals and the prevalence of these effects in the population (Ince et al., 2021).

The extent to which these biases negatively influence everyday decisions remains unclear, although repeating previous choices in situations of uncertainty may serve to preserve neural resources associated with choice alternation and to maintain self-consistency (Peters et al., 2017). Indeed, activation of a specific cortical network involving inferior frontal cortex and the subthalamic nucleus during the decision process is associated with disengagement from choice hysteresis (Fleming, Thomas, & Dolan, 2010). This suggests that switching choices under conditions of uncertainty comes at a computational cost. It is interesting to speculate that engagement of this network might improve performance but not subjective confidence in the choice, thereby explaining the lack of metacognitive insight our participants displayed, despite increased perceptual sensitivity, when alternating from their previous choice (Figures 5C–E). Furthermore, the drive for hysteresis/self-consistency may induce uncertainty when choices are switched and, hence, distort metacognitive judgements.

It is possible that such biases could have negative implications in circumstances where significant decisions must be made under conditions of high uncertainty (i.e., security scanning, medical imaging [Bruno, Walker, & Abujudeh, 2015]). Furthermore, miscalibrated metacognitive judgement (systematic under- or overconfidence) is likely to impact on learning, adaptive decision-making, and mental health (van den Berg et al., 2016; Desender et al., 2018; Yeung & Summerfield, 2012; Bahrami et al., 2012; Folke et al., 2016; Rouault et al., 2018; Benwell et al., 2022), and may be compounded by history and confirmation biases. The development of behavioral or pharmacological techniques to reduce such biases can help to optimize accurate decision-making and self-reflection.

## Conclusion

Choice history independently influences both perceptual decisions and subjective confidence ratings in humans, resulting in suboptimal perceptual and metacognitive sensitivity and highlighting dissociation of decision-monitoring processes from the decisions themselves.

## Supplementary Material

Supplement 1
