# Supplementary material for: History biases reveal novel dissociations between perceptual and metacognitive decision-making
Source: J Vis. 2023 May 18;23(5):14. doi: 10.1167/jov.23.5.14 (PMC10207958; doi:10.1167/jov.23.5.14)
Supplement: Supplement 1 [file jovi-23-5-14_s001.pdf]

Supplementary Information

History biases reveal novel dissociations between perceptual and metacognitive decision-making

Christopher S.Y. Benwell[1,2], Rachael Beyer[2], Francis Wallington[2], Robin Ince[2]

[1] Division of Psychology, School of Humanities, Social Sciences and Law, University of Dundee, Dundee, UK

[2] School of Psychology and Neuroscience, University of Glasgow, Glasgow, UK

Supplementary Figures

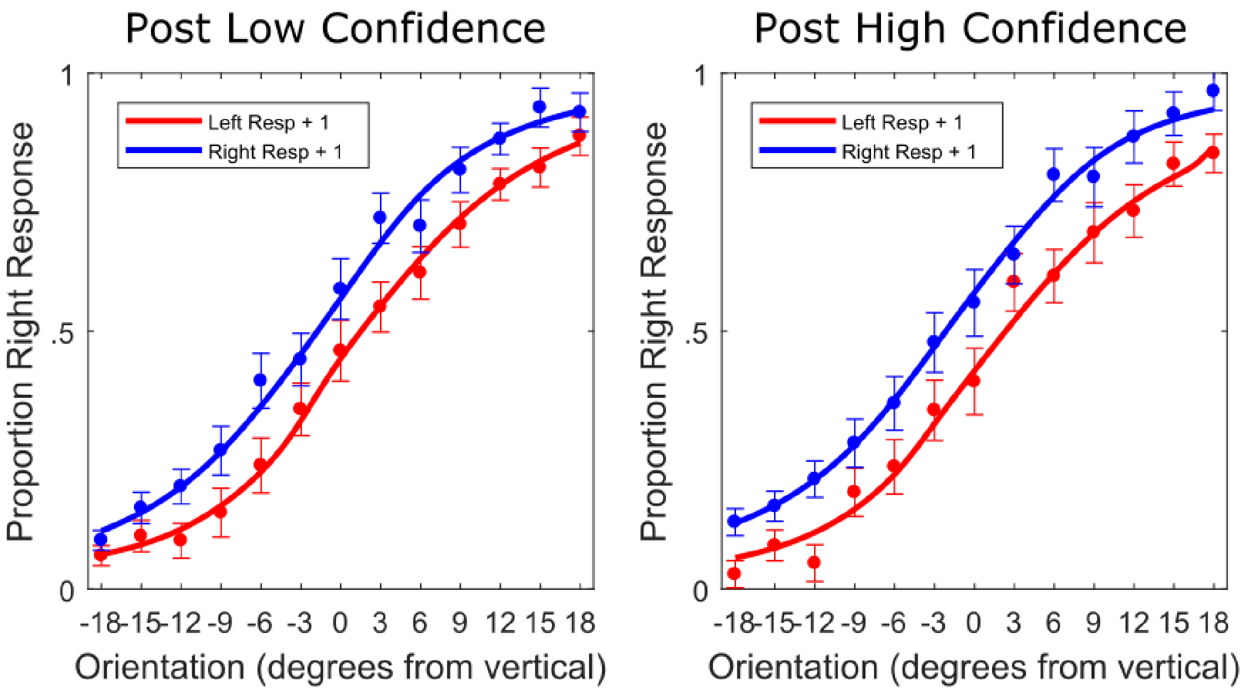

Supplementary Figure 1. Prior confidence did not significantly alter the strength of perceptual choice history bias. The same figure as Figure 2A but separately for ‘post low confidence’ and ‘post high confidence’ trials. Trials were split into post high and low confidence bins as evenly as possible within each participant (see Methods). Four participants had to be excluded because they did not have enough trials in one of the conditions to reliably retrieve psychometric function (PF) parameter estimates (see Methods). A 2 (previous confidence level: low versus high) x 2 (previous perceptual choice: left versus right) repeated-measures ANOVA on PF thresholds revealed a significant main effect of previous perceptual choice (F(1,32) = 11.584, p = .002, η_p^2 = .266), no main effect of previous confidence level (F(1,32) = .891, p = .352, η_p^2 = .027) and no interaction between the two (F(1,32) = 1.689, p = .203, η_p^2 = .05). Post ‘left choice’ thresholds were significantly different to post ‘right choice’ thresholds, both for post low confidence trials (t(32) = 4.029, p < .001, BF_{10} = 88.338) and for post high confidence trials (t(32) = 2.781, p = .009, BF_{10} = 4.765). The difference in perceptual choice history bias (post ‘left choice’ – post ‘right choice’ thresholds) between post high and post

low confidence trials was not significant (t(32) = 1.3, p = .203, BF_{10} = 0.402). These results are in apparent contrast to those of previous studies (Urai et al., 2017; Braun et al., 2018; Suarez-Pinilla et al., 2018; Samaha et al., 2019; Bosch et al., 2020). For instance, Braun et al., (2018) showed that confidence (indexed by RT and accuracy) boosted adaptation to experimentally controlled changes in the repetition probability of the stimuli. Here we did not manipulate repetition probability which remained random throughout. We also employed explicit reports of confidence whereas previous studies have employed RT, accuracy and/or pupil diameter as proxies for confidence/uncertainty (Urai et al., 2017; Braun et al., 2018). In another study involving explicit reports, Samaha et al., (2019) employed a task which dissociated confidence ratings from objective performance and found that when sensory evidence in favour of one choice versus another was kept constant, higher confidence trials led to stronger serial dependence than lower confidence trials. Hence, 'pure' fluctuations of confidence occurring irrespective of relative choice evidence may influence history bias (Samaha et al., 2019), whereas the effect may be ameliorated when most ratings are informed by the quality of evidence (as was the case here). In other words, the interplay between perceptual decisions, confidence and choice history bias might vary depending on the information available to inform decisions and confidence judgements (Bosch et al., 2020). This hypothesis could be directly tested by manipulating the quality of evidence available for perceptual choices whilst matching task accuracy (Samaha et al., 2019; Desender et al., 2018) within different blocks of the same experiment. It would also be instructive for future studies to tease apart and compare the relative contributions of different measures which putatively index uncertainty/confidence (such as explicit ratings, RT, event-related potentials (Gherman & Philiastides, 2015; Murphy et al., 2015) and pupil diameter) but which may exert dissociable influences on perceptual and metacognitive history biases (Urai et al., 2017).

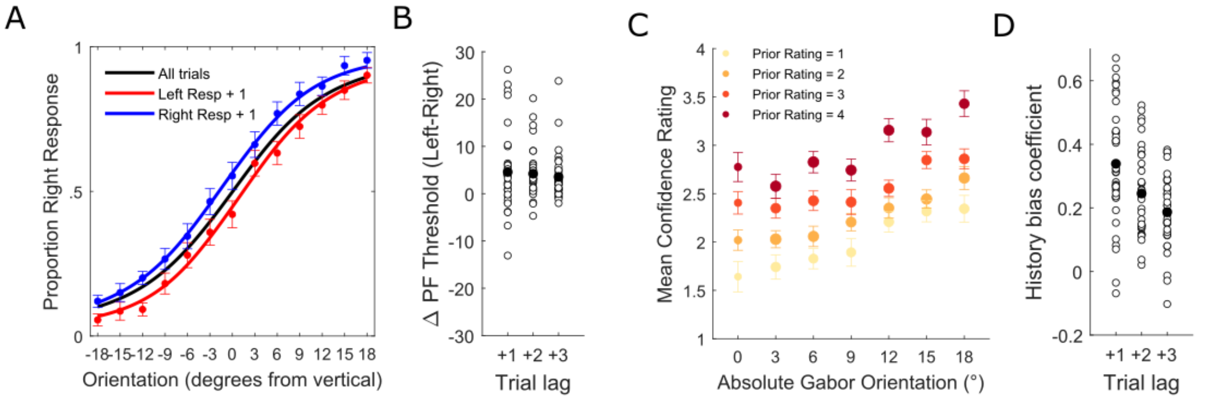

Supplementary Figure 2. When conditioning the trial splits post-correct trials only (**a**), post-left PF thresholds were significantly biased away from veridical 0° (t(36) = 2.4413, p = .0197, BF10= 2.3673), as were post-right PF thresholds, but in the opposite direction (t(36) = -2.3379, p = .0251, BF10 = 1,9359). Accordingly, post-left thresholds were significantly different to post-right thresholds (t(36) = 3.4294, p = .0015, BF10 = 21.4131). (**b**) The effect remained significant for trial lags of two (t(36) = 5.086, p < .001, BF10 = 1.7861e+03) and three (t(36) = 4.5496, p < .001, BF10 = 400.7977). (**c**) A regression analysis confirmed that confidence was positively predicted by ratings on the previous trial across participants (t-test of slopes versus 0: t(36) = 11.0914, p < .001, BF10 = 2.1653e+10). (**d**) The effect remained significant for trial lags of two (t(36) = 11.0751, p < .001, BF10 = 2.0814e+10) and three (t(36) = 10.2509, p < .001, BF10 = 2.7098e+09).

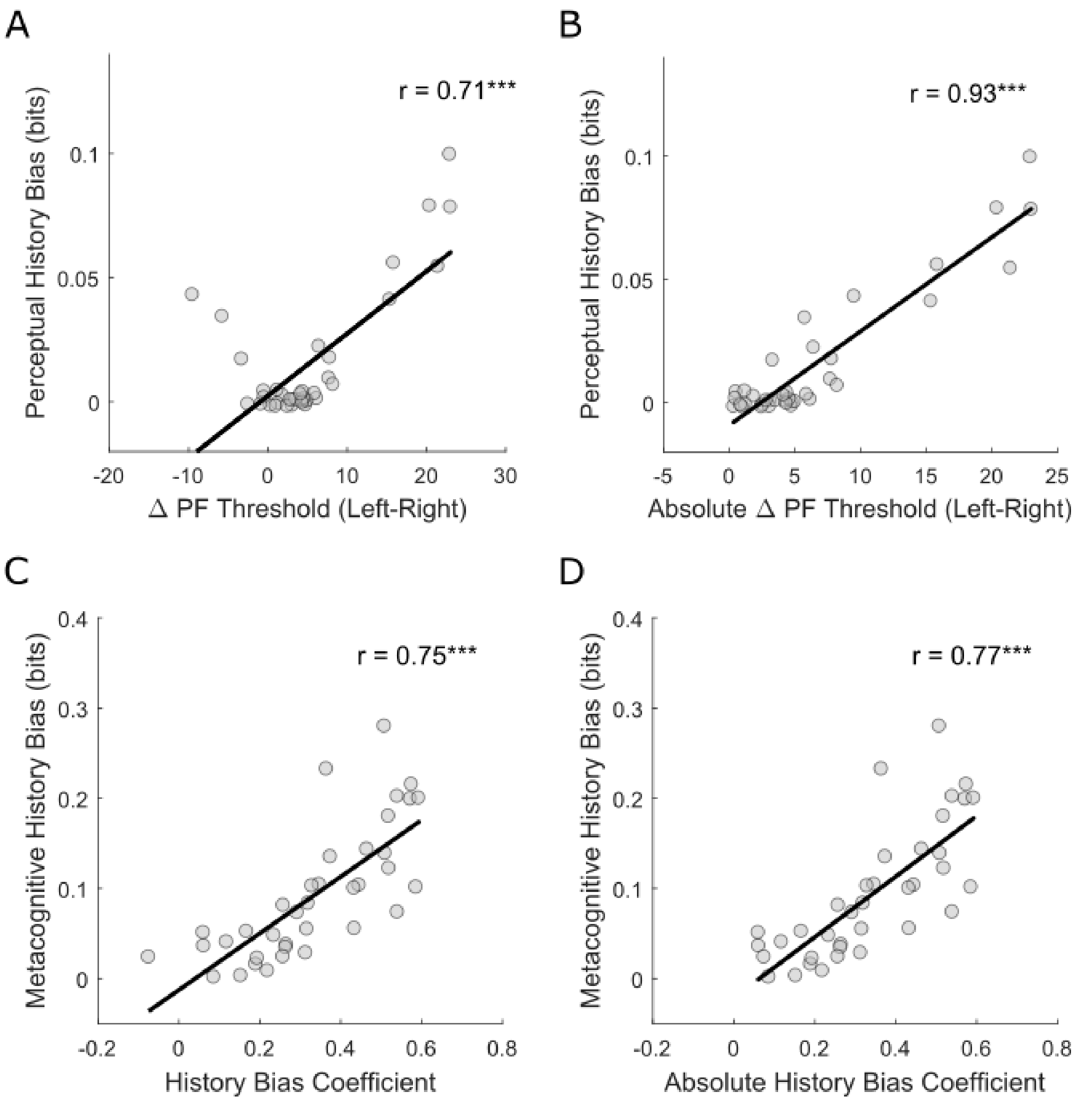

Supplementary Figure 3. Correlations between model-based and non-parametric measures of choice history bias. The MI history bias measure is an assumption free measure which indexes how strongly current responses are related to previous responses, irrespective of the evidence available on a given trial. **(a)** Relationship between differences in psychometric function (PF) threshold between 'post-left choice' and 'post-right choice' trials and perceptual history bias quantified using mutual information (I(resp-1;resp)) (Pearson's $r = 0.7124$, $p < .001$, Spearman's rho = 0.4697, $p = .004$, $BF_{10} = 2.3173e+04$). Notice the expected u-shaped relationship which occurs because MI is an unsigned quantification of dependence (i.e., it does not dissociate 'repetition' from 'alternation' biases). **(b)** Relationship between absolute differences in psychometric function (PF) threshold between 'post-left choice' and 'post-right choice' trials and perceptual history bias quantified using mutual information (I(resp-

1;resp)) (Pearson's r = 0.9292, $p < .001$, Spearman's rho = 0.725, $p < .001$, BF_{10} = 6.9779e+13). The absolute strength of the PF difference bias (regardless of sign) is strongly linearly related to the MI measure of bias. **(c)** Relationship between metacognitive history bias coefficients and metacognitive history bias quantified using mutual information (I(conf-1;conf)) (Pearson's r = 0.7516, $p < .001$, Spearman's rho = 0.8286, $p < .001$, BF_{10} = 1.9176e+05). **(d)** Relationship between absolute metacognitive history bias coefficients and metacognitive history bias quantified using mutual information (I(conf-1;conf)) (Pearson's r = 0.7654, $p < .001$, Spearman's rho = 0.8257, $p < .001$, BF_{10} = 4.4338e+05).

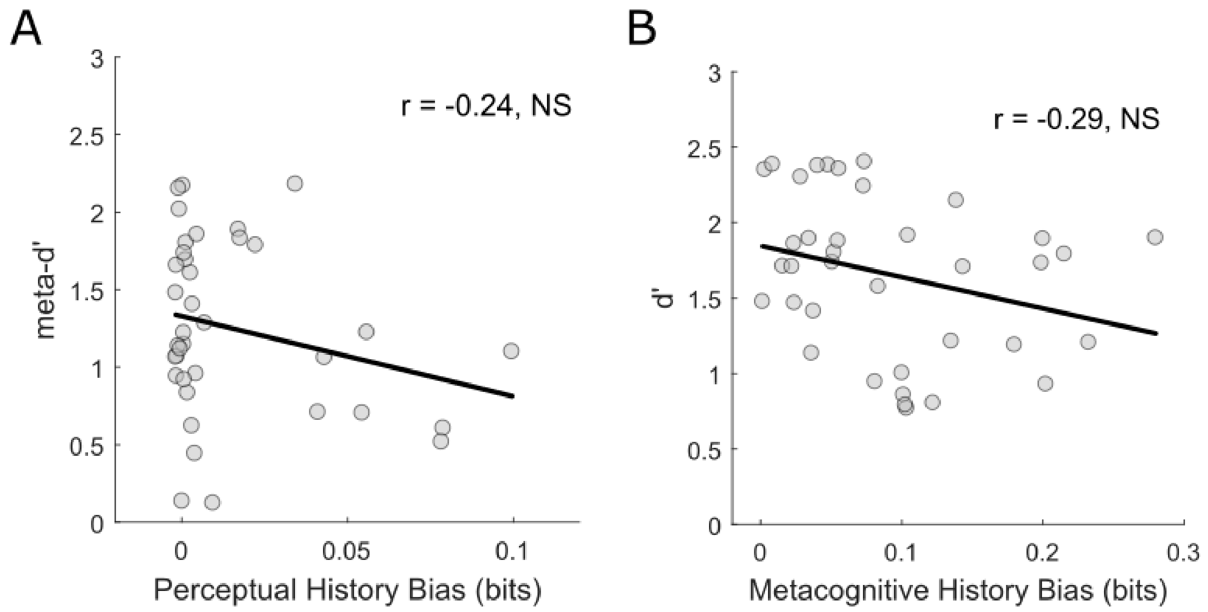

Supplementary Figure 4. Between-subjects Pearson correlations. **(a)** Relationship between perceptual choice history bias and metacognitive sensitivity (***meta-d'***) (Pearson's r = -0.245, p = .1439, Spearman's rho = -0.1802, p = .2847, BF_{10} = 0.369). **(b)** Relationship between metacognitive choice history bias and perceptual sensitivity *(**d'***) (Pearson's r = -0.2874, p = .0846, Spearman's rho = -0.2992, p = .0724, BF_{10} = 0.56).

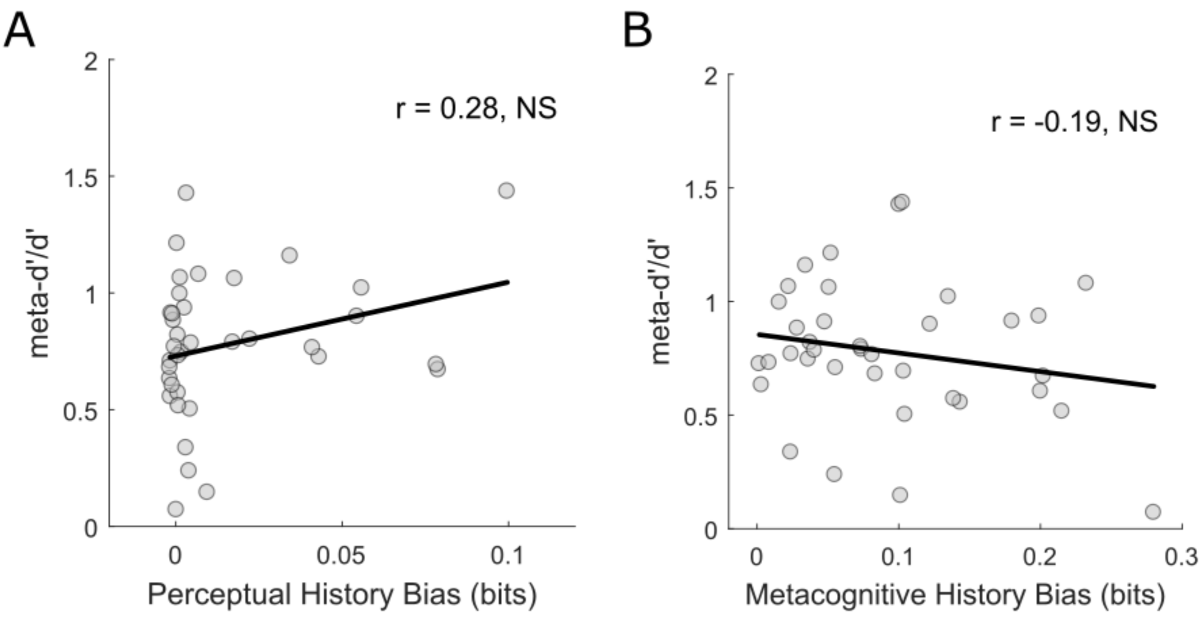

Supplementary Figure 5. Between-subjects metacognitive efficiency ratio correlations. **(a)** Relationship between perceptual choice history bias and metacognitive efficiency (***meta-d'/d'***) (Pearson's r = 0.2759, p = .0983, Spearman's rho = 0.2349, p = .1612, BF_{10} = 0.497). **(b)** Relationship between metacognitive choice history bias and metacognitive efficiency (***meta-d'/d'***) (Pearson's r = -0.1944, p = .249, Spearman's rho = -0.169, p = .316, BF_{10} = 0.247).

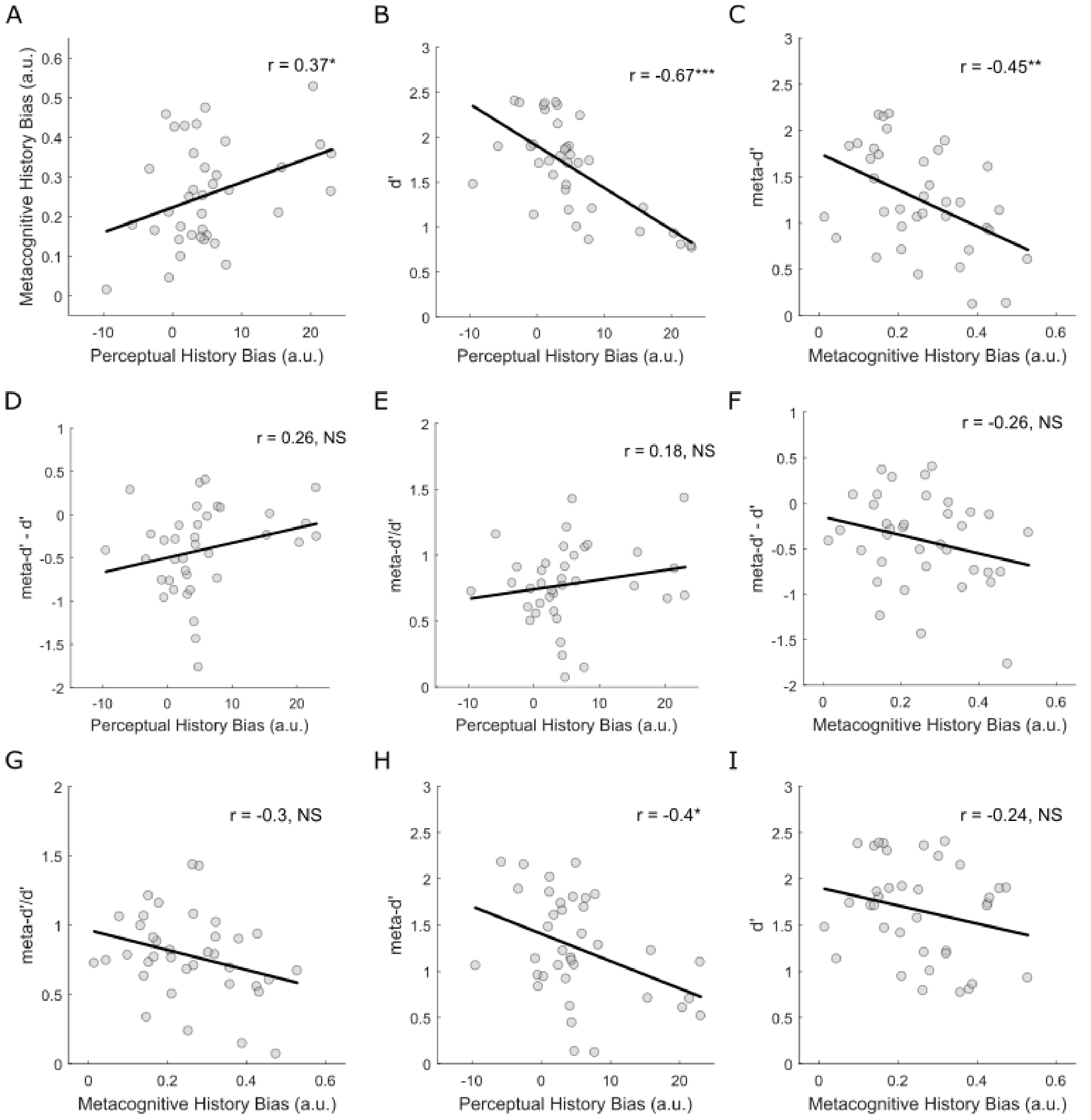

Supplementary Figure 6. Between-subjects Pearson correlations using metrics of choice history bias which retain the direction of bias (i.e., repeater (+ve x-axis values) versus alternator (-ve x-axis values)). Perceptual choice history bias was quantified for each participant by subtracting their post left choice PF threshold from their post right choice PF threshold. Metacognitive choice history bias was quantified by a linear regression coefficient linking confidence ratings on trials 1:415 and confidence ratings on trials 2:416. **(a)** Relationship between perceptual and metacognitive choice history biases (Pearson's r = 0.3723, p = .0232, Spearman's rho = 0.2667, p = .1105, BF_{10} = 1.647). The positive correlation suggests that the stronger the positive perceptual history bias (i.e., 'repeaters'), the

stronger the metacognitive choice history bias. However, though the Pearson correlation was significant, Spearman's Rho was not significant and BF_{10} indicated that the data were insensitive as to whether the effect exists. Hence, this effect should be treated with caution, and we do not interpret it further here. **(b)** Relationship between perceptual choice history bias and perceptual sensitivity ***(d')*** (Pearson's r = -0.6725, p < .001, Spearman's rho = -0.6385, p < .001, BF_{10} = 3.7753e+03). Stronger perceptual repetition biases were associated with reduced perceptual sensitivity. **(c)** Relationship between metacognitive choice history bias and metacognitive sensitivity (***meta-d'***) (Pearson's r = -0.4525, p = .005, Spearman's rho = -0.426, p = .009, BF_{10} = 6.462). Stronger metacognitive repetition biases were associated with reduced metacognitive sensitivity. **(d)** Relationship between perceptual choice history bias and metacognitive efficiency (indexed by ***meta-d' – d'***) (Pearson's r = 0.26, p = .12, Spearman's rho = -0.3872, p = .018, BF_{10} = 0.425). The positive correlation suggests that the stronger the positive perceptual history bias (i.e., 'repeaters'), the higher the level of metacognitive insight. However, though the Spearman correlation was significant, Pearson's *r* was not significant and BF_{10} indicated that the data were insensitive as to whether the effect exists. **(e)** Relationship between perceptual choice history bias and metacognitive efficiency (indexed by ***meta-d'/d'***) (Pearson's r = 0.18, p = .285, Spearman's rho = 0.2378, p = .156, BF_{10} = 0.2255). **(f)** Relationship between metacognitive choice history bias and metacognitive efficiency (indexed by ***meta-d' – d'***) (Pearson's r = -0.26, p = .1198, Spearman's rho = -0.2278, p = .1745, BF_{10} = 0.4252). **(g)** Relationship between metacognitive choice history bias and metacognitive efficiency (indexed by ***meta-d'/d'***) (Pearson's r = -0.3031, p = .0682, Spearman's rho = -0.3025, p = .0691, BF_{10} = 0.6662). **(h)** Relationship between perceptual choice history bias and metacognitive sensitivity (***meta-d'***) (Pearson's r = -0.3978, p = .0148, Spearman's rho = -0.3227, p = .0519, BF_{10} = 2.4422). The negative correlation suggests that the stronger the perceptual repetition bias, the lower the metacognitive sensitivity. However, though the Pearson correlation was significant, Spearman's Rho was not significant and BF_{10}indicated that the data were insensitive as to whether the effect exists. **(i)** Relationship between metacognitive choice

history bias and perceptual sensitivity ***(d')*** (Pearson's r = -0.2388, p = .1546, Spearman's rho = -0.2148, p = .2010, BF_{10} = 0.3498).

Supplementary References

1 Urai, A. E., Braun, A. & Donner, T. H. Pupil-linked arousal is driven by decision uncertainty and alters serial choice bias. *Nature communications* **8**, 14637, doi:10.1038/ncomms14637 (2017).

2 Braun, A., Urai, A. E. & Donner, T. H. Adaptive History Biases Result from Confidence-weighted Accumulation of Past Choices. *The Journal of neuroscience : the official journal of the Society for Neuroscience*, doi:10.1523/JNEUROSCI.2189-17.2017 (2018).

3 Suarez-Pinilla, M., Seth, A. K. & Roseboom, W. Serial dependence in the perception of visual variance. *J Vision* **18**, doi:Artn 4 10.1167/18.7.4 (2018).

4 Samaha, J., Switzky, M. & Postle, B. R. Confidence boosts serial dependence in orientation estimation. *J Vision* **19**, doi:Artn 25 10.1167/19.4.25 (2019).

5 Desender, K., Boldt, A. & Yeung, N. Subjective Confidence Predicts Information Seeking in Decision Making. *Psychological science* **29**, 761-778, doi:10.1177/0956797617744771 (2018).

6 Gherman, S. & Philiastides, M. G. Neural representations of confidence emerge from the process of decision formation during perceptual choices. *NeuroImage* **106**, 134-143, doi:10.1016/j.neuroimage.2014.11.036 (2015).

7 Murphy, P. R., Robertson, I. H., Harty, S. & O'Connell, R. G. Neural evidence accumulation persists after choice to inform metacognitive judgments. *eLife* **4**, doi:ARTN e1194610.7554/eLife.11946 (2015).
